# Dysregulated MicroRNAs as Biomarkers or Therapeutic Targets in Cisplatin-Induced Nephrotoxicity: A Systematic Review

**DOI:** 10.3390/ijms222312765

**Published:** 2021-11-25

**Authors:** Nadine de Godoy Torso, João Kleber Novais Pereira, Marília Berlofa Visacri, Pedro Eduardo Nascimento Silva Vasconcelos, Pía Loren, Kathleen Saavedra, Nicolás Saavedra, Luis A. Salazar, Patricia Moriel

**Affiliations:** 1 School of Medical Sciences, University of Campinas, Campinas 13083894, Brazil; nadinetorso@gmail.com (N.d.G.T.); joaoknpereira@gmail.com (J.K.N.P.); mariberlofa@gmail.com (M.B.V.); pedro.nsvasconcelos89@gmail.com (P.E.N.S.V.); 2 Center of Molecular Biology and Pharmacogenetics, Scientific and Technological Bioresource Nucleus, Universidad de La Frontera, Temuco 4811230, Chile; pia.loren@ufrontera.cl (P.L.); kathleen.saavedra@ufrontera.cl (K.S.); nicolas.saavedra@ufrontera.cl (N.S.); luis.salazar@ufrontera.cl (L.A.S.); 3 Faculty of Pharmaceutical Sciences, University of Campinas, Campinas 13083970, Brazil

**Keywords:** cisplatin, drug-related side effects and adverse reactions, acute kidney injury, microRNAs, epigenomics, biomarkers, systematic review

## Abstract

The purpose of this systematic review was to map out and summarize scientific evidence on dysregulated microRNAs (miRNAs) that can be possible biomarkers or therapeutic targets for cisplatin nephrotoxicity and have already been tested in humans, animals, or cells. In addition, an in silico analysis of the two miRNAs found to be dysregulated in the majority of studies was performed. A literature search was performed using eight databases for studies published up to 4 July 2021. Two independent reviewers selected the studies and extracted the data; disagreements were resolved by a third and fourth reviewers. A total of 1002 records were identified, of which 30 met the eligibility criteria. All studies were published in English and reported between 2010 and 2021. The main findings were as follows: (a) miR-34a and miR-21 were the main miRNAs identified by the studies as possible biomarkers and therapeutic targets of cisplatin nephrotoxicity; (b) the in silico analysis revealed 124 and 131 different strongly validated targets for miR-34a and miR-21, respectively; and (c) studies in humans remain scarce.

## 1. Introduction

Cisplatin is an antineoplastic agent widely used in the treatment of solid tumors because of its extensive cytotoxic activity; however, it is associated with a high incidence of treatment-induced acute kidney injury (AKI) [1]. The kidney plays a crucial role in the excretion of drugs and is therefore highly susceptible to drug use-related injury. The known mechanisms underlying cisplatin-induced nephrotoxicity include apoptosis, autophagy, nuclear and mitochondrial DNA damage and production of reactive oxygen species [2]. Because it is dose-limiting, the dose of cisplatin is often reduced or treatment discontinued following nephrotoxicity, which can increase the risk of disease recurrence or progression.

The biomarkers used routinely to assess kidney function and damage are mainly serum creatinine (CRE), blood urea nitrogen (BUN), and urine output levels. However, these biomarkers have low sensitivity and specificity for detecting early events associated with AKI [3,4] and are significantly altered only after the marked progression of kidney injury [5]. A late diagnosis impairs the timely treatment of patients with AKI, which is also an aggravating factor in several oncological treatments based on cisplatin regimens. Therefore, identification of new nephrotoxicity biomarkers is urgently required.

MicroRNAs (miRNAs) have attracted considerable research attention as novel biomarkers. miRNAs are a class of non-coding RNAs that are approximately 20 bp in length and involved in gene regulation at the post-transcriptional level. miRNAs regulate gene expression by base pairing with sequences located in the 3′-untranslated regions on their respective target messenger RNAs (mRNAs) [6,7]. They are involved in both physiological and pathological processes, playing different roles in cell growth and differentiation, apoptosis, tumorigenesis [8,9,10], and embryogenesis [11]. As the AKI is a complex syndrome with different possible etiologies [12], studies involving miRNAs and different causes of AKI have been previously published in order to discover new sensitive and predictive biomarkers for this syndrome [13]. A mouse study conducted by Bellinger et al. [14] examined the changes in miRNAs expression after bilateral ischemia-reperfusion injury, which has been shown to cause miR-714, miR-1188, miR-1897-3p, and miR-1224 elevation in plasma and kidneys. Other studies also point to miR-10a as an increased miRNA in plasma of rats with renal ischemia-reperfusion injury [15] and in serum samples of patients diagnosed with AKI [16]. Furthermore, the administration of an miR-10a agonist in rats intensified the level of cell apoptosis in the renal tissues, while the administration of an miR-10a antagonist attenuated the renal injury [17].

The potential application of miRNAs as biomarkers stems from their high conservation between species, specific expression in different tissues or pathological states [18], the relatively simple laboratory methods used for their analysis, and their stability in different body fluids (e.g., blood and urine, commonly used as samples because they can be collected using minimally invasive procedures) [19]. Typically, necrotic or apoptotic cell death due to lesions culminates in the release of miRNAs into biofluids [20,21]. Therefore, in clinical practice, these biomarkers can be used as AKI predictors, diagnostic biomarkers (identifying patients with or without AKI), or AKI severity biomarkers.

The possibility of using miRNAs in therapeutics exists because of their ability to target multiple mRNAs, and thus, regulate multiple genes. The therapeutic approach involves two possibilities: the use of therapeutic miRNAs, including the use of miRNA mimics to restore the expression of those that are repressed, and the use of miRNAs as therapeutic targets, including the use of inhibitors of miRNA inhibitors (anti-miRs) to reduce the expression of the miRNAs upregulated during pathogenesis [22].

Therefore, as epigenetic modulators, the identification of changes in miRNA expression in response to AKI induced by cisplatin may provide new candidates for biomarkers and therapeutics. The purpose of this systematic review was to map out and summarize scientific evidence regarding miRNAs that are potential biomarkers or therapeutic targets of cisplatin nephrotoxicity that have already been tested in humans, animals, or cells. In addition, an in silico analysis of the two miRNAs found to be dysregulated in the majority of studies was performed.

## 2. Methods

This review followed the recommendations of the Preferred Reporting Items for Systematic Reviews and Meta-Analyses Statement for Scoping Reviews (PRISMA-ScR) [23], and the review protocol was registered in Open Science Framework (https://doi.org/10.17605/OSF.IO/M5WV2, last update on 15 December 2020). 

### 2.1. Search Strategy

The literature search was conducted using PubMed, PubMed Central, BIREME, Web of Science, Scopus, Embase, Cochrane Library, and EBSCOhost databases to retrieve pertinent studies published up to 4 July 2021, without language restriction. The search strategy encompassed terms related to cisplatin, nephrotoxicity, and miRNAs, and the complete search strategy used in each database can be found in File S1. To ensure search sensitivity, the authors selected four articles a priori, which were all recovered by the search.

### 2.2. Study Selection

Studies were eligible for this systematic (scoping) review if they addressed the role of miRNAs as possible biomarkers and therapeutic targets of cisplatin-induced nephrotoxicity in cells, animals, or humans. Only original articles that evaluated the expression of miRNAs following exposure to cisplatin, not concomitantly with another drug, as well as those studies that comprised some nephrotoxicity assessment method, quantified miRNA expression or showed these data, and did not have an exclusive computational approach (“in silico”) were included in this review. No language restrictions were imposed.

Duplicate studies retrieved using the search strategy were excluded using EndNote^®^. The remaining studies were allocated to the Rayyan^®^ tool (https://www.rayyan.ai/, accessed on 12 July 2021), where two review steps were performed: screening and analysis of the article titles and abstracts. This last step was performed independently by two reviewers (NGT and JKNP), and any discrepancies were discussed with a third and fourth reviewer (PM and MBV).

The studies selected for the first analysis were moved to the complete and critical article reading stage, which was also performed by two reviewers independently (NGT and JKNP), and the discrepancies were discussed with a third and fourth reviewer (PM and MBV). In addition, references cited in all included articles were reviewed to identify any studies that might have been missed in the search.

### 2.3. Data Extraction and Analysis

Data extracted from articles that met the requirements included author, year of publication, country, aim of the study, study population (cell, animal, or human), cisplatin treatment/exposure (dose/concentration), nephrotoxicity assessment, methods used to identify miRNAs, time of the analysis related to cisplatin exposure, miRNAs significantly differentially expressed in nephrotoxicity, pathophysiological implications of dysregulated miRNAs (only those related to the authors’ own results and discussions; results or discussions based on other studies were not collected), and role of miRNAs in nephrotoxicity. The extracted data were entered into a Microsoft Word spreadsheet^®^, and this process was again conducted by two independent reviewers (NGT and JKNP), and disagreements were discussed with a third and fourth reviewer (PM and MBV). The included studies were categorized according to the studied population, and the results of this review were presented using the narrative synthesis approach. 

### 2.4. Bioinformatics Analysis

To generate the interaction network of selected miRNAs (miR-34a and miR-21), we employed miRTargetLink 2.0, a tool containing experimentally validated interactions of human miRNA-mRNA pairs. The data shown correspond to strongly supported miRNAtarget interactions, that is, those validated experimentally using reporter assays, western blotting, RT-qPCR, microarray, or next-generation sequencing experiments. miRNA annotations were obtained from the latest version of miRBase (v.22.1), while the experimentally validated targets were retrieved from miRTarBase (v.8) and miRATBase. miRTargetLink 2.0 is freely accessible (https://ccbcompute.cs.uni-saarland.de/mirtargetlink2/, accessed on 26 August 2021).

## 3. Results and Discussion

**Figure 1:**
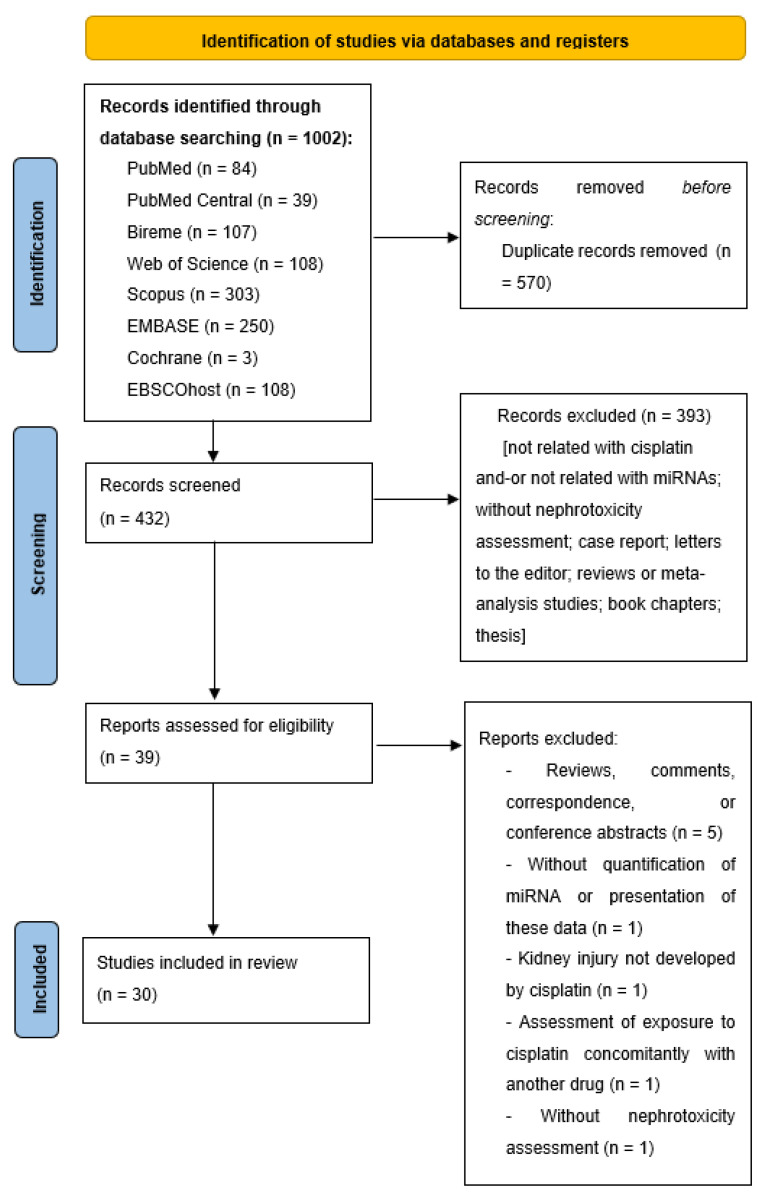
Study selection flow diagram used for the literature search.

The search strategy identified 1002 studies from multiple databases, with 570 overlapping studies. Of the remaining 432 articles, 393 were excluded by reviewing their titles and abstracts. The full text of the remaining 39 articles underwent a review, and 30 studies [24,25,26,27,28,29,30,31,32,33,34,35,36,37,38,39,40,41,42,43,44,45,46,47,48,49,50,51,52,53] that met the eligibility criteria were included. Figure 1 shows a flowchart of the literature search and Supplementary Table S1. ontains a reference list of the studies excluded in the full-text review, along with the reasons for their exclusion.

All studies were published in English, between 2010 and 2021. Most of these studies were conducted in China [24,25,31,32,36,37,38,39,40,41,42,43,44,47,48,49,50] and the United States of America (USA) [26,27,34,38,46]. The main differences among the 30 included studies were related to population, cisplatin dose, nephrotoxicity assessment, and time of analysis of the miRNAs. Seven studies were conducted only in cells [24,25,36,47,48,49,50], 11 only in animal models [26,27,28,29,30,31,32,33,51,52,53], 10 in both in vitro and in vivo models [34,35,37,38,39,40,41,42,43,44], one in cells and humans [46], and only one in humans [45]. The principal methods used for assessing nephrotoxicity in the studies conducted in cells were viability and apoptosis tests, with 17 studies using at least one of these. Of the 21 studies involving animal models, only four did not assess nephrotoxicity by histological analyses [28,34,38,39], and only three did not measure BUN or CRE levels [28,37,53]. Human studies used two different criteria to assess nephrotoxicity, but both were based on serum CRE levels; one used the Common Toxicity Criteria for Adverse Events (version 4) [45], while the other used the AKI Network criteria [46]. As for the cisplatin treatment used, neither the dose nor the duration of exposure or treatment was similar among the studies. A summary of the characteristics of the 30 studies included in this systematic review is presented in Table 1.

### 3.1. Methods of Included Studies

For miRNA identification, the cellular studies used cells or the supernatants of cell cultures, and only one did not specify the sample used [48]. Animal studies used plasma, urine, or kidney tissue, while one human study used plasma samples [45] and the other used urine samples [46]. Regardless of the population studied, the method chosen for miRNA identification was mainly real-time polymerase chain reaction (RT-PCR), which in some cases was also associated with microarray or miRNA-seq techniques. RT-PCR is the gold standard method for quantifying miRNAs, and the results of studies that did not use RT-PCR should be validated using this technique.

In addition, all studies evaluated the expression of miRNAs after exposure to cisplatin, although the exact time was different for each of them. The study by Quintanilha et al. was the only one to propose miRNA as a predictor of nephrotoxicity, as they identified altered expression of miRNAs in patients with nephrotoxicity before chemotherapy with cisplatin [45]. Table 2 presents the main results of the included studies.

Interestingly, some studies selected miRNAs for validation based on their sequencing or array results, while others selected miRNAs based on the existing literature and on the action of target genes already known for these miRNAs. 

Findings from in vitro models are important for discovering potential therapeutic targets; however, as these findings may differ from those of other organisms, it is essential that they be validated in in vivo models.

The in vivo model studies included in this review used serum, urine, or renal tissue samples, and those performed in humans used plasma or urine. Blood contains high levels of potentially interfering proteins, and alterations in serum biomarker levels may not be related to renal function, as it can be a systemic response. Instead, urine samples have several advantages, such as non-invasive collection in large quantities and strong specificity for kidney damage [55].

Although extracellular fluids may contain RNA-degrading enzymes, miRNAs remain stable and detectable in blood because they are associated with protein complexes [56]; in addition, they are present inside exosomes [57]. Urinary miRNAs appear to be derived from the kidney and urinary tract cells and are filtered through the glomerulus or secreted by renal tubules [58]. 

### 3.2. miRNAs as Biomarkers or Therapeutic Agents or Targets of AKI

**Figure 2:**
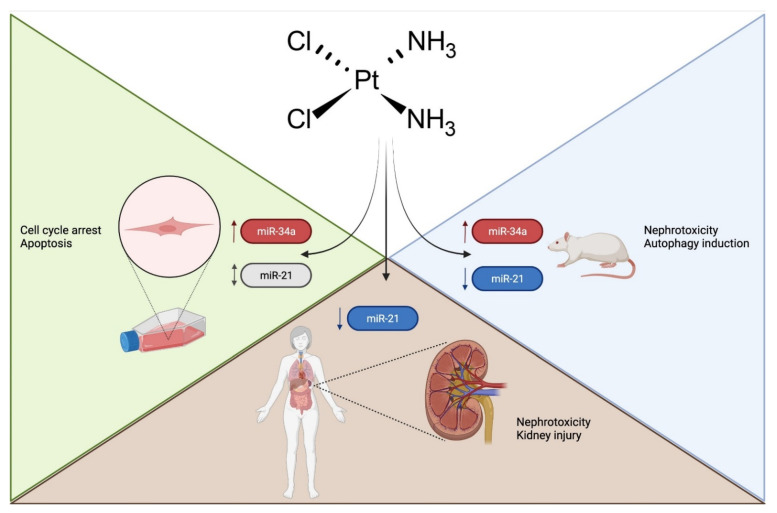
Involvement of the two miRNAs that appeared as dysregulated miRNAs in most of the studies included in this review. The blue color indicates downregulation of gene expression; the red color indicates upregulation of gene expression, and the black color indicates inconsistencies in the literature. Created with BioRender.com, accessed on 4 October 2021.

A total of 115 different miRNAs were found to be differentially expressed in the studies included in this systematic review (Supplementary Table S2), with 90 being dysregulated in one study and only 25 in more than one (Table 3). The miRNAs that most frequently appeared in the studies were miR-34a and miR-21, appearing in six and five studies, respectively (Figure 2). miR-34a was upregulated in all six studies included [27,30,34,35,47,52], and miR-21 was upregulated in four studies [33,46,52] and downregulated in one study [25]. 

Disregarding that some miRNAs were cited more than once, this review identified 63 miRNAs proposed by the included articles as possible new biomarkers: 23 by Kanki et al. [51], 19 by Pavkovic et al. [52], 10 by Harrill et al. [26], four by Suter-Dick et al. [47], three by Pavkovic et al. [46], three by Quintanilha et al. [45], two by Kagawa et al. [29], and one each by Cho et al. [53], Wolenski et al. [27], Glineur et al. [28], and Xiong et al. [44]. Other studies, such as those of Qin et al. [36], Jiang et al. [48], Zhang et al. [50], Hao et al. [38], Liao et al. [39], Yang et al. [41], and Xiong et al. [44], proposed that dysregulated miRNAs could serve as therapeutic targets or agents of cisplatin-induced AKI.

Some miRNAs, including miR-181a [24,31], miR-192 [47,52], miR-122 [35], miR-26a [35,42], miR-30a-e [37], miR-375 [38], miR-140-5p [39], miR-709 [40], miR-199a-3p [41], and miR-31 [32], have been evaluated in different in vivo and in vitro models.

By comparing the nephrotoxicity profile presented by different individuals, animals, or cells undergoing the same treatment with cisplatin, it is possible to identify specific miRNA profiles, which can, therefore, be exploited to overcome diagnostic and therapeutic challenges. In this case, it is important to differentiate that some miRNAs were identified by the included studies as possible biomarkers of AKI, and others were identified as possible therapeutic targets (in accordance with the purpose of each study). Despite this, many miRNAs can function as biomarkers as well as potential targets. For example, the detection of miRNAs expressed in blood as AKI biomarkers could serve as a basis for the administration of a mimic miRNA or an anti-miR, regulating the pathway that is dysregulated in AKI, thus, functioning as a potential treatment.

The early detection of cisplatin-induced AKI is essential in the case of patients with cancer receiving cisplatin-based treatments. To this end, new biomarkers with adequate efficiency and sensitivity are needed. As miR-34a and miR-21 were the miRNAs that were the most frequently reported for this possible use in previous studies (six and five citations in different studies, respectively), the current discussion mainly focuses on them. 

The use of miRNAs in cisplatin nephrotoxicity therapy aims to provide new approaches for the treatment of AKI through which miRNAs can be upregulated if they are related to anti-necroptotic or anti-inflammatory pathways, or they can be silenced if they activate pro-apoptotic genes. However, the ability of a single miRNA to regulate multiple genes in signaling pathways can be both beneficial and harmful. Caution is needed regarding off-target effects because there is a possibility that an anti-miR can inhibit other miRNAs with a common seed region [59]. Furthermore, since various miRNAs can target the same mRNA, the identification and validation of the downstream targets of miRNAs are unclear. Lastly, this therapeutic tool has some barriers, including the potential for degradation by RNases as well as the need for an efficient in vivo delivery system [22].

miR-34a was upregulated in all of the six included studies, whether it was in cells or animal urine and kidneys [27,30,34,35,47,52]. Unlike other members of the miR-34 family, miR-34a is ubiquitously expressed in normal human tissues. It is a p53-target gene, implying that miR-34a expression is transcriptionally regulated by p53; however, some studies have shown that miR-34a levels can be regulated by p53-independent mechanisms [60]. 

The induction of this miRNA by p53 targeting during DNA damage promotes apoptosis and cell cycle arrest [60,61]. Moreover, p53 activation is one of the mechanisms that contribute to renal cell death during cisplatin nephrotoxicity [62].

In a study by Lee et al., miR-34a was upregulated in the kidney tissue of mice after cisplatin treatment. This increase promoted the acetylation of Foxo3 by repressing the expression of SIRT1, an NAD-dependent deacetylase involved in the control of pro-apoptotic protein synthesis [63]. It was shown to be a bridge to p53-dependent apoptosis, and miR-34a is involved in this network controlling cisplatin-induced tubular injury. Indeed, the in silico analysis of the current study confirmed the prediction of SIRT1 as one of the most predicted targets of miR-34a. 

The study by Lee et al. also showed two antagonistic mechanisms of apoptosis modulation during cisplatin treatment: (1) treatment with an miR-34a mimic enhanced the acetylation of Foxo3 and promoted the expression of p53 and Bax (a mediator of mitochondria-dependent programmed cell death), decreasing cell viability and (2) the miR-34a-antisense oligonucleotide transfection antagonized the ability of cisplatin to increase p53 and Bax levels and improved cell viability compared with the controls [35]. 

The activation of the miR-34a and p53 networks in response to cisplatin was also reported in a study by Pavkovic et al., where miR-34a was found to be upregulated, along with 17 other miRNAs, in rat urine over 26 days of investigation after cisplatin exposure [52]. The mRNA-predicted target analysis of these miRNAs was associated with various mechanisms known to be involved in cisplatin-induced nephrotoxicity [62,64], such as DNA damage response, apoptosis, cell cycle regulation, and inflammation [52]. 

Moreover, El Magdoub et al. showed the involvement of another pathway. They found that cisplatin treatment in rats upregulated miRNA-34a and induced the expression of transforming growth factor beta (TGF-β), a mediator of renal fibrosis that affects kidney function by stimulating extracellular matrix protein production [65]. As a consequence, TGFβR-1 was also induced and led to the stimulation of TAK1 [30]. Interestingly, TAK1 regulates cell necroptosis and has already been reported to regulate nuclear factor kappa B production [66], which was also elevated after cisplatin treatment [65].

However, a study by Bhatt et al. showed that blocking the induction of miR-34a with antisense oligonucleotides increased cell death during cisplatin treatment and exacerbated tissue damage, suggesting that this miRNA may play a cytoprotective role [34]. They also confirmed the involvement of p53 in miR-34a upregulation by cisplatin in cells treated with pifithrin-α (a pharmacological inhibitor of p53) as well as in p53-deficient mice. In both cases, the induction of miR-34a expression by cisplatin was completely or largely suppressed [34].

This difference between the findings of these studies (some indicating a protective role and others indicating nephrotoxicity induction) shows that the role of miR-34a in cisplatin-induced AKI is still controversial. One of the challenges in identifying new miRNA biomarkers is that many studies have reported conflicting data.

miR-34a was primarily identified by the studies included in this systematic review as a potential AKI biomarker; nevertheless, the therapeutic use of miR-34a has already been investigated in a phase I clinical trial. As it is a known tumor suppressor that is not highly expressed in most malignancies, the trial treated solid malignancies, refractory to standard treatment, with an miR-34a mimic delivered intravenously through liposomes [67]. However, the study reported adverse effects and could not confirm whether they were related to the miR-34a mimic or the liposomal carrier. This raises concerns regarding the effects of miR-34a or other miRNA-mediated modulation strategies on AKI treatment.

miR-21 was the second miRNA whose possible use as a biomarker of cisplatin-induced AKI was the most reported in previous studies. miR-21 is considered a promising biomarker for AKI because its expression is conserved in the kidneys [68], an organ in which it performs physiological and pathological functions [69]. Specific to the studies included in this systematic review, miR-21 was upregulated in four studies [33,46,52] and downregulated in one study [25]. 

In a study by Pavkovic et al., miR-21 levels increased in the cellular medium and decreased in the proximal kidney tubular epithelial cells. This change has been suggested as a hypothesis to explain the presence of this and other miRNAs in urine [46]. This is also the case for miR-15, miR-16, miR-20a, miR-192, miR-193, and miR- 210, which were found to be upregulated in urine samples and downregulated in kidney tissue samples [52].

Various studies that did not involve cisplatin but that also evaluated the role of miR-21 as an AKI biomarker have previously been carried out. In a study by Du et al., in adult patients undergoing cardiac surgery, urinary and plasma miR-21 levels were associated with severe AKI [70]. In humans, increased urinary miR-21 expression has been associated with the progression of renal inflammation and fibrosis [71]. A third study using models of diabetic nephropathy showed that animals transfected with miR-21 knockdown plasmids showed improvement in microalbuminuria, fibrosis, and renal inflammation [72].

Contrastingly, some studies have shown that when miR-21 is upregulated in AKI, it plays a protective role by inhibiting the apoptosis and necrosis of renal tubular epithelial cells in response to stress in kidney injury renal ischemia-reperfusion [73,74]. Furthermore, it has been demonstrated to have therapeutic potential for the inhibition of fibrosis in pulmonary tissue by decreasing miR-21 levels [75].

Therefore, the results obtained to date indicate that the role of miR-21 must be associated with a high-precision adjustment mechanism to maintain its physiological equilibrium. Low miR-21 expression results in increased cell death, as miR-21 acts as an anti-apoptotic agent, while the overexpression of miR-21 can lead to severe inflammation and fibrosis [18].

Importantly, neither of the two miRNAs (miR-34a and miR-21) had their results validated in human cohorts. This reveals a gap in the literature, as results found in in vivo and in vitro models will not necessarily be similar to those observed in humans. 

In silico analysis revealed 124 and 131 different strongly validated targets for miR-34a and miR-21, respectively (targets validated through robust experiments that, in addition to gene expression, evaluated protein expression and function, using analyses such as luciferase reporter assay and western blotting). Most of the targets were predicted by more than one type of experiment and more than one study. 

For miR-34a, the most predicted targets were *BCL2*, *MET*, *NOTCH1*, and *SIRT1*; for miR-21-5p, they were *PDCD4*, *PTEN*, and *RECK*. Based on the results of this analysis, Supplementary Figures S1 and S2 were prepared for the two miRNAs, showing the relationships of their targets with various possible pathways involved in cisplatin nephrotoxicity.

*BCL2* and *SIRT1* have been shown to be involved in apoptosis regulation pathways, which are key processes in the pathophysiology of cisplatin nephrotoxicity [62]. In contrast, the *MET* and *NOTCH1* genes, despite having been detected as possible targets, were not related to these selected pathways or the other two genes; they are involved in oncogenesis [76] and the development of numerous cell and tissue types [77].

As for miR-21, the main predicted targets were *PDCD4*, *PTEN*, and *RECK*. The *PDCD4* and *PTEN* genes are involved in the regulation of apoptosis, the mitogen-activated protein kinase cascade, and protein phosphorylation; all of these processes are related to cisplatin nephrotoxicity [78]. The *RECK* gene, which is not related to any of these pathways, is mainly involved in embryogenesis and vasculogenesis [79].

### 3.3. Future Perspectives

One of the challenges in developing miRNAs biomarkers is the high cost of the techniques for miRNAs detection; therefore, more studies should be carried out on their cost-effectiveness and cost-utility, evaluating the possible benefits of early care with the use of these miRNAs, either by improving the quality of life or the outcome of cisplatin treatment. 

Furthermore, those miRNAs that showed good prospects in animal trials still have a long way to go before being implemented in the clinic, which includes having their results validated in humans and having sensitivity and specificity at least comparable to those of currently used biomarkers. In addition, a set of miRNAs is more likely to be more robust than a single miRNA [80].

Once these described implementation challenges are overcome, they could be useful in different contexts: they could be used in pretreatment tests (as a predictor of nephrotoxicity induced by cisplatin) or in post-treatment tests (as early markers that would detect nephrotoxicity earlier than currently used biomarkers). 

## 4. Conclusions

The data presented in the studies included in this review show that miR-34a and miR-21 appear to be promising new biomarkers for cisplatin-induced nephrotoxicity. Despite this, validation studies in humans are still needed for the use of these miRNAs in clinical practice.

## Figures and Tables

**Table 1:** Characteristics of the studies included in the systematic review.

**Cells**					
**Author(s)/Year**	**Country**	**Aim**	**Population**	**Cisplatin Treatment/Exposure**	**Nephrotoxicity Markers**
Zhu et al./2012 [24]	China	To investigate the role of miR-181a in the apoptosis of tubular epithelial cell induced by CIS.	HK-2 cells.	50 µmol/L for 24 h.	Apoptosis.
Zhang et al./2015 [25]	China	To elucidate the role of miR-205 in CIS-induced renal cell apoptosis and explored the molecular mechanisms.	HK-2 cells.	100 μg/mL for 6 h.	Apoptosis.
Jiang et al./2019 [48]	China	To evaluate miRNAs that potentially target MLKL and evaluate their function in human tubular epithelial cells in response to toxic and ischemic insults.	HK-2 cells.	20 μM	Programmed cell death and the relative mRNA levels of KIM-1.
Wu et al./2019 [49]	China	To examine the changes of the miRNA and mRNA expression profiles in CIS treated HK-2 cells.	HK-2 cells.	10 µM for 24 h.	Cell viability and apoptosis.
Zhang et al./2020 [50]	China	To improve the therapeutic efficacy in CIS-induced AKI.	HK-2 cells.	15 µM for up to 48 h.	Apoptosis, inflammatory markers (TNF-a and IL-8) and necroptosis.
Suter-Dick et al./2018 [47]	Switzerland	To investigate changes in miRNA released to the cell culture medium as potential markers for nephrotoxicity.	HPTECs cells line overexpressing the OAT1.	0–30 μM for 24 h or 48 h.	Cell viability and NAG.
Qin et al./2016 [36]	China	To explore the expression and function of miR-449 in CIS-induced AKI.	NRK-52E cells.	20 μg/mL for 24 h.	Cell viability and apoptosis.
**Animals**					
**Author(s)/Year**	**Country**	**Aim**	**Population**	**Cisplatin Treatment/Exposure**	**Nephrotoxicity Markers**
Cho et al./2017 [53]	Republic of Korea	To evaluate if miRNAs circulating exosomes may serve as biomarkers of drug-induced kidney injury.	Male Balb/C mice.	Single injection, 10 mg/kg, IP.	Histological analyses.
Huang et al./2020 [31]	China	To investigate the role of miRNAs against CIS-induced AKI in mice.	Male C57BL/6 mice	20 mg/kg/day, for three, IP.	BUN and histological analyses
Harrill et al./2017 [26]	USA	To investigate performance of urinary kidney biomarkers against classical preclinical kidney injury biomarkers.	Female DO mice	Single injection, 5 mg/kg, IP.	BUN, serum CRE, histological analyses, and urine b2-microglobulin, interferon gamma-induced protein 10, KIM-1, renin, and osteopontin.
Glineur et al./2018 [28]	Belgica	To evaluate the performance of a panel of 68 urinary miRNAs as potential nephron segment-specific biomarkers before and after treatment with nephrotoxic drugs.	Male Hannover Wistar rats.	Single dose, 2.5 mg/kg, IP.	Urinary KIM-1, albumin, and clusterin, and histological analyses.
Kanki et al./2014 [51]	Japan	To identify urinary miRNAs markers suitable for use in detecting CIS-induced nephrotoxicity.	Male Sprague-Dawley rats.	Study 1: single injection, 6 mg/kg, IP. Study 2: single injection, 0, 1, 3 and 6 mg/kg, IP.	BUN, serum CRE, urinary KIM-1 and clusterin, and histopathological analysis.
Wolenski et al./2017 [27]	USA	To dose rats with toxicants that cause nephrotoxicity, and then identify changes within the miRNA expression profiles in urine, plasma, and tissue.	Male Sprague-Dawley rats	Single dose, 2 or 5 mg/kg, IV.	Serum BUN, urinary CRE and KIM-1, and histological analyses.
Kagawa et al./2019 [29]	Japan	To identify plasma miRNAs that may enable early and specific detection of drug-induced tubular and glomerular injury through next-generation sequencing analysis.	Male Sprague-Dawley rats.	Single dose, 6 mg/kg, IV.	Plasma CRE and BUN, urinary albumin, and histological analyses.
Wu et al./2020 [32]	China	To investigate the function of puerarin in a CIS-induced AKI rat model via RT-qPCR and western blot analyses.	Female Sprague-Dawley rats.	20 mg/kg, IP.	BUN, serum CRE, and histological analyses.
Pavkovic et al./2014 [52]	Germany	To evaluate whether urinary miRNAs could serve as biomarkers for CIS-induced kidney injury.	Male Wistar rats.	Single dose, 1 or 3 mg/kg, IP.	BUN, serum CRE, urinary KIM-1 and alpha-GST, and histopathological analysis.
El Magdoub et al./2020 [30]	Egypt	To investigate the involvement of miRNAs let-7b, 26b, and 34a on CIS-induced nephrotoxicity.	Male Wistar rats.	Single dose on day 4, 5 mg/kg, IP.	Plasma urea, CRE and KIM-1, and histological analyses.
Okamoto et al./2021 [33]	Japan	To investigate clinicopathological changes in dogs treated with a clinical dose of CIS.	Female beagle dogs	70 mg/m^2^ over 20 min, IV (via cephalic vein).	Urinary protein, CRE, NAG, albumin, and L-FABP; blood CRE, BUN, magnesium, calcium, inorganic phosphorus, sodium, potassium, and chloride; histopathological analysis.
**Cells and Animals**					
**Author(s)/Year**	**Country**	**Aim**	**Population**	**Cisplatin Treatment/exposure**	**Nephrotoxicity Markers**
Bhatt et al./2010 [34]	USA	To examine the regulation of miR-34a and its role in CIS-induced AKI and nephrotoxicity.	BUMPT-306 cells.	40 μmol/L for 0–12 h.	Apoptosis.
			Male wild type and p53-deficient C57BL/6 mice.	Single injection of CIS 30 mg/kg, IP.	BUN.
Du et al./2017 [37]	China	To investigate the mechanism of CIS-induced renal injury.	HK-2 and NRK-52E cells.	10 μM for 12, 24, 48 or 72 h.	Apoptosis.
			Wistar rats.	10 mg/kg, IP.	Apoptosis and histological analyses.
Liao et al./2017 [39]	China	To investigate the levels of miR-140-5p and its functional role in pathogenesis of CIS-induced AKI.	HK-2 cells.	20 μM for 6 h.	ROS and LDH levels, MnSOD activity, and cell viability.
			Male mice.	Single injection, 20 mg/kg, IP.	BUN and serum CRE.
Yang et al./2019 [41]	China	To probe into the role of p53 in CIS-induced AKI.	HK-2 cells.	100 μM for 24 h.	Apoptosis.
			C57/BL male mice.	Single dose, 20mg/kg, IP.	BUN, serum CRE, and histological analyses.
Yang et al./2019 [42]	China	To study on whether miR-26a plays an anti-apoptotic role through regulating the expression of TRPC6.	HK-2 cells.	0, 1, 2, 4 or 8 μM.	Apoptosis.
			Male C57BL/6 mice.	20 mg/kg, IP.	BUN, serum CRE, and histological analyses
Li et al./2021 [43]	China	To examine the role of long noncoding RNA PRNCR1 in CIS-induced AKI in vitro and in vivo.	HK-2 cells.	10^−9^, 10^−8^, 10^−7^ and 10^−6^ M for 24 h.	Cell viability and apoptosis.
			Male BALB/c mice.	20 mg/kg, IP.	Serum CRE and histological analyses.
Guo et al./2018 [40]	China	To investigate the pathogenic role of miR-709 in mediating mitochondrial impairment and tubular cell death in AKI.	mPTC cells.	0–20 mM CIS for 0–24 h.	Apoptosis and determination of mitochondrial function.
			Male C57BL/6 mice.	20 mg/kg, IP.	BUN, serum CRE, histological analyses, and determination of mitochondrial function.
Lee et al./2014 [35]	Korea	To evaluate an integrative network of miRNAs and mRNA data to discover a possible master regulator of AKI.	NRK-52E cells.	30 mmol/L for 48 h.	Cell viability.
			Male C57BL/6 mice.	Single dose, 15 mg/kg, IP.	BUN, serum CRE, KIM-1, neutrophil elastase, F4/80, CD3e, CD19, TNF alfa, ICAM-1, and histopathological and immunohistochemical analysis.
Xiong et al./2021 [44]	China	To investigate the roles of miR-186 in cisplatin-induced AKI.	NRK-52E cells.	6 μM.	Cell viability and apoptosis.
			Male Wistar rats.	6 mg/kg, IP.	Plasma BUN and CRE, and histological analyses.
Hao et al./2017 [38]	USA andChina	To examine the role of miRNAs in CIS-induced nephrotoxicity.	RPTC cells.	20 μM for 16 h.	Apoptosis.
			Male C57 mice.	30 mg/kg, IP.	BUN and serum CRE.
**Humans**					
**Author(s)/Year**	**Country**	**Aim**	**Population**	**Cisplatin Treatment/Exposure**	**Nephrotoxicity Markers**
Quintanilha et al./2021 [45]	Brazil	To identify circulating plasma miRNAs as biomarkers of cisplatin-induced nephrotoxicity using the patients’ samples.	Patients with primary squamous cell carcinoma of the head and neck.	One cycle of 80 or 100 mg/m^2^.	Serum CRE, BUN, CRE clearance, and Common Toxicity Criteria for Adverse Events version 4.
**Humans and Cells**					
**Author(s)/Year**	**Country**	**Aim**	**Population**	**Cisplatin Treatment/Exposure**	**Nephrotoxicity Markers**
Pavkovic et al./2016 [46]	USA	To evaluate KIM-1 in conjunction with miRNAs as biomarkers for drug-induced AKI.	HPTEC cells.	85 μM, for 24 h.	Cell viability.
			Patients with malignant mesothelioma.	Intraoperative CIS therapy.	AKI defined by AKI Network criteria (serum CRE dependent status) [54].

Abbreviations: AKI, acute kidney injury; alpha-GST, alpha glutathione s-transferase; BUMPT-306, mouse proximal tubular cell; BUN, blood urea nitrogen; CRE, creatinine; CIS, cisplatin; HK-2, human kidney 2; HPTECs, primary human proximal tubular epithelial cells; ICAM-1, intercellular adhesion molecule-1; IV, intravenous administration; IP, intraperitoneal administration; KIM-1, Kidney Injury Molecule-1; LDH, lactate dehydrogenase; L-FABP, liver-type fatty acid binding protein; MLKL, mixed lineage kinase domain-like; MnSOD, manganese superoxide dismutase; mPTC, mouse proximal tubular cells; NAG, N-acetil-beta-D-glucosaminidase; NR, not reported; NRK-52E, rat proximal tubule epithelial cells; OAT1, organic anion transporter 1; PTEN, phosphatase and tensin homolog; ROS, reactive oxygen species; RPTC, rat proximal tubular cells; RT-qPCR, real-Time quantitative polymerase chain reaction; TRPC6, target transient receptor potential channel 6.

**Table 2:** miRNAs as biomarkers and therapeutic targets of nephrotoxicity in the studies included in the systematic review.

**Cells**						
**Author(s)/Year**	**Sample**	**Methods Used to Identify miRNAs**	**Time When the miRNAs Were Analyzed** **(after CIS Exposure/Administration)**	**miRNAs Differentially Expressed in Nephrotoxicity**	**Pathophysiological Implications of Dysregulated miRNAs** **(Related to the Authors’ Own Results)**	**Role of miRNAs as Biomarkers of Nephrotoxicity**
Zhu et al./2012 [24]	Cells (HK-2)	RT-qPCR	24 h	Upregulated: miR-181a	CIS upregulated miR-181a expression leading to negative regulation of Bcl-2 (anti-apoptotic gene) and positive regulation of BAX (pro-apoptotic gene). Thus, miR-181a expression is associated with cell apoptosis.	CIS may play a role in tubular epithelial cell apoptosis by suppressing Bcl-2 expression, which is achieved by regulating the target gene of microRNA-181a. These findings pave a novel approach to the enhancement of prevention treatment of CIS-induced nephrotoxicity.
Zhang et al./2015 [25]	Cells (HK-2)	RT-qPCR	6 h	Downregulated: miR-205 miR-21 miR-29	MiR-205 could be an anti-apoptotic molecule, because it coordinates the expression of this target gene CMTM4 to module the renal cells apoptosis.	MiR-205 was revealed as an important inhibitor in the regulation of apoptosis in renal cells.
Jiang et al./2019 [48]	NR	RT-PCR	12, 24, 36, and 48 h	a. Upregulated: miR-194-5p miR-577 b. Downregulated: miR-500a-3p	Overexpression of miR-500a-3P had a protective role in CIS-induced kidney injury, as it showed: - To limit programmed cell death; - To decreased chemokine MCP-1 and proinflammatory cytokines TNF-a and IL-8; - To decrease phosphorylation and membrane translocation of MLKL, a key index for detecting necroptosis.	Considering the antinecroptotic and anti-inflammatory merits, miR-500a-3P may be a novel therapeutic agent for AKI treatment.
Wu et al./2019 [49]	Cells (HK-2)	MiRNA microarray and RT-qPCR	24 h	1. Microarray results: 26 miRNAs upregulated and 21 miRNAs downregulated. Top 5: a. Upregulated: miR-4793-3p miR-4299 miR-4440 miR-297 miR-4485 b. Downregulated: miR- 6841-5p miR-3605-5p miR-27b-5p miR-1236-5p miR-23b-5p 2. RT-qPCR results (validation of 5 miRNAs): Upregulated: miR-4299 miR- 297 miR- 3135b miR-9-3p miR-371b-5p	HIPK2, a key regulator of kidney fibrosis, was predicted as the common target gene of miR-9-3p and miR-371b-5p.	An integrative network approach encompassing miRNAs, target genes, and bioinformatics analysis showed that miR-9-3p and miR-371b-5p could be critical miRNAs in CIS-induced renal tubular cell injury.
Zhang et al./2020 [50]	Cells (HK-2)	RT-qPCR	6, 12, 24, 36, and 48 h	Downregulated: miR-500a-3P	The downregulation of miR-500a-3P in CIS-induced kidney injury was related to apoptosis, NF-kB-based inflammation (increased expression of TNF-a, IL-8, and P-P65), and necroptosis by RIPK3-based downstream signaling pathway (increased expression of P-MLKL and RIPK3 proteins).	MiR-500a-3P is effective in controlling the AKI and may be an appropriate miRNA therapeutics.
Suter-Dick et al./2018 [47]	Supernatant of cells culture (HPTECs cells line overexpressing the OAT1).	RT-PCR	0, 24, and 48 h	Upregulated: Cells treated with 5 uM CIS in 48h: miR-34a miR-192 miR-29a miR-21 Cells treated with 15 uM CIS in 24h: miR-34a miR-192 miR-29a	No explanations were provided.	The data suggest that mir-21, mir-29a, mir-34a and mir-192 are early and sensitive biomarkers of damage to renal proximal tubule cells.
Qin et al./2016 [36]	Cells (NRK-52E)	RT-qPCR	72 h	Upregulated: miR-449	SIRT1/P53/BAX pathway is related with apoptosis. The study results showed that SIRT1 was inhibited and acetylated p53 and BAX were promoted when miR-449 was upregulated by CIS. Thus, miR-499 expression inhibits cell viability and intensifies apoptosis.	The data suggest that there may exist a pro-apoptotic role of miR-449 in CIS-induced AKI via regulating the SIRT1/P53/BAX pathway. Therefore, it is suggested that miR-449 be a potential therapeutic target for treating AKI.
**Animals**						
**Author(s)/Year**	**Sample**	**Methods Used to Identify miRNAs**	**Time When the miRNAs Were Analyzed** **(after CIS Exposure/Administration)**	**miRNAs Differentially Expressed in Nephrotoxicity**	**Pathophysiological Implications of Dysregulated miRNAs** **(Related to the Authors’ Own Results)**	**Role of miRNAs as Biomarkers of Nephrotoxicity**
Cho et al./2017 [53]	Plasma.	RT-qPCR.	NR	Upregulated: miR-146a	No explanations were provided.	MiR-146a is a potential biomarker for drug-induced kidney injury.
Huang et al./2020 [31]	Kidney	RT-qPCR	Day 3.	Upregulated: miR-181a	MiR-181a can be involved in inhibit PTEN protein expression.	PTEN is one of the miR-181a targets.
Harrill et al./2017 [26]	Urine.	Microarray with RT-qPCR.	54 h.	Of the 335 unique miRNAs assayed on the array platform, 10 miRNA species were significantly affected by treatment (all upregulated) and differed in abundance based on presence of tubule necrosis (grade 2): miR-130a miR-151-3p miR-218 miR-320 miR-680 miR-138 miR-152 miR-221 miR-328 miR-685	No explanations were provided.	These urinary miRNAs are biomarker candidates, as they were elevated alongside BUN and protein biomarkers in animals with grade 2 proximal tubular cell necrosis.
Glineur et al./2018 [28]	Urine	PCR	Before and days 1, 3, 6, 13, 20, and 27 after.	FC > 3.0-Upregulated: miR-34c-5p FC < 3.0-Upregulated: miR-183-5p miR-15b-5p miR-193a-3p miR-423-3p miR-182-5p miR-210-3p miR-155-5p	Urinary miR-34c-5p after CIS administration probably reveals CIS-induced DNA damage leading to p53 transcription factor activation. Moreover, the miR-34c-5p urinary release could attest the activation of a cellular apoptotic process. miR-34c-5p outperformed KIM-1 under subacute conditions (100% of accurate classification vs 75% with KIM-1).	MiR-34c-5p could complement urinary protein biomarkers to detect subacute CIS-induced kidney injury.
Kanki et al./2014 [51]	Urine and kidney tissue.	Microarray and RT-qPCR.	*Study 1:* day 5. Study 2: days 1, 3 and 7.	Levels of 25 miRNAs were elevated in urine concurrently with the appearance of the necrosis of proximal tubules (most of them were conversely decreased in the cortex or outer medulla of kidney): miR-335 miR-378a-5p miR-183-5p miR-328a-3p miR-1839-5p let-7a-1-3p miR-93-5p miR-532-3p miR-192-5p miR-20b-5p miR-17-5p miR-140-3p miR-25-3p miR-340-5p miR-191a-5p let-7g-5p miR-193-5p miR-218a-5p miR-7a-1-3p miR-130b-3p miR-30a-5p miR-26b-3p miR-744-5p miR-320-3p	No explanations were provided.	These 25 urinary miRNAs biomarkers could potentially be used to detect proximal tubular injury due to CIS exposure. They were considered to have sensitivities comparable to BUN, serum CRE, and urinary protein biomarkers.
Wolenski et al./2017 [27]	Plasma, urine, and kidney tissue.	NGS	24 and 72 h.	FC > 2,0: 1. Kidney tissue (upregulated): miR-34a 2. Urine (upregulated): miR-378a miR-1839 miR-140 miR-26b let-7g 3.Plasma (upregulated): miR-34c miR-128 miR-34a miR-130b	No explanations were provided.	MiR-378a is a novel urinary biomarker of kidney damage.
Kagawa et al./2019 [29]	Plasma	NGS and RT-qPCR	Days 0, 1, 2, 3, and 5.	1. Early, downregulated: miR-122-5p miR-143-3p miR-26a-5p miR-215 let-7c-5p miR-146b-5p miR-192-5p miR-191a-5p 2. Middle, downregulated: miR-122-5p miR-143-3p let-7i-5p miR-140-3p let-7g-5p let-7f-5p miR-378a-3p miR-148a-3p miR-25-3p miR-23a-3p 3. Late, downregulated: miR-122-5p miR-143-3p let-7i-5p miR-140-3p let-7g-5p let-7f-5p miR-148a-3p miR-23a-3p miR-26a-5p miR-215 let-7c-5p miR-30a-5p miR-486	No explanations were provided.	MiR-143-3p and miR-122-5p may be potential biomarkers for the early detection of tubular damage. The downregulation of these miRNAs was earlier than the changes in the traditional biomarkers, such as plasma CRE and BUN.
Wu et al./2020 [32]	Kidney	RT-qPCR	Days 0, 1, 3, and 5.	Upregulated: miR-31	The study hypothesized that the negative regulation of Numb affects Notch signaling via miR-31 in CIS-induced AKI, because Notch signaling is associated with the balance among the cell proliferation and apoptosis that influence the process of various organ injuries.	MiR-31 expression is upregulated in CIS-induced AKI.
Pavkovic et al./2014 [52]	Kidney tissue and urine.	Microarray with RT-qPCR.	Kidney tissue: days 3, 5, 8, and 26. Urine: days 3, 5, 8, 15, and 26.	1. Urine: a. CIS 3mg/kg: 136 miRNAS appeared significantly affected by CIS. 11 upregulated selected by >20-fold change: miR-15b miR-16 miR-20a miR-20b miR-21 miR-34a miR-185 miR-192 miR-200b miR-210 miR-339-3p b. CIS 1mg/kg or 3 mg/kg (day 3 and day 5). Upregulated: miR-15 miR-16 miR-20a miR-192 miR-193 miR-210 2. Tissue: a. CIS 1 mg/Kg. Upregulated: miR-21 miR-34a miR-184 miR-327 b. Donwregulated: miR-15b miR-16 miR-20a miR-20b miR-141 miR-146a miR-185 miR-192 miR-193 miR-196c.rno miR-200b miR-210 miR-223	These miRNAs are associated with pathways, as DNA damage response, apoptosis, cell cycle regulation, and inflammation. The top canonical pathway affected were p53 and PI3K/AKT pathways. Also, mRNAs predicted as targets of the altered miRNAs in the kidney were associated with DNA damage response, apoptosis, and cell cycle regulation.	These miRNAs are potential urinary biomarkers for CIS-induced kidney injury.
El Magdoub et al./2020 [30]	Kidney tissue	RT-PCR	Day 3.	Downregulated: miRNA-let-7b miRNA-26b *Upregulated:* miRNA-34a	With the alteration of these miRNAs expressions, CIS induced TGF-β1. TGFβR-1, TAK1, and mTOR levels were increased, while LC3-II level was decreased.	Potential involvement of those 3 miRNAs in the pathogenesis of CIS-induced nephrotoxicity.
Okamoto et al./2021 [33]	Serum and urine	RT-qPCR	0, 6, 12, 24, 72, and 168 h.	Serum-Upregulated: miR-21 miR-26a miR-10a Urine-Upregulated: miR-21	Serum miR-21 was correlated with dynamics of blood calcium, inorganic phosphorus, and magnesium. Increased serum miR-21 levels might indicate kidney injury, as well as biological alerts from a damaged vascular system or other organs.	Increased serum miR-21 levels might indicate kidney injury. Altered urinary levels of miR-21 and miR-26a (or miR-10a) might reflect tubulointerstitial and glomerular lesions by CIS, respectively.
**Cells and Animals**						
**Author(s)/Year**	**Sample**	**Methods Used to Identify miRNAs**	**Time When the miRNAs Were Analyzed** **(after CIS Exposure/Administration)**	**miRNAs Differentially Expressed in Nephrotoxicity**	**Pathophysiological Implications of Dysregulated miRNAs** **(Related to the Authors’ Own Results)**	**Role of miRNAs as Biomarkers of Nephrotoxicity**
Bhatt et al./2010 [34]	Cells (BUMPT-306)	RT-qPCR	0, 4, 8, and 12 h.	Upregulated: miR-34a	This study showed that miR-34a induction during CIS nephrotoxicity was mediated by p53. However, blockage of miR-34a increased cell injury and death. The authors speculated that miR-34a may regulate or repress proapoptotic genes.	There is evidence for a cytoprotective role of induced miR-34a against CIS-induced apoptosis in renal cells.
	Kidney tissue		Days 0, 1, 2, and 3.			
Du et al./2017 [37]	Cells (HK-2 and NRK-52E).	RT-qPCR	24, 48, 72 h (HK2 cells) or 12, 24, 48 h (NRK-52E).	Downregulated-Both cells: miR-30a miR-30b miR-30c miR-30d miR-30e	The downregulation of miR-30c induced by CIS positively regulated the expression of its Bnip3L and Hspa5 target genes, which resulted in significant increase on apoptosis.	MiR-30c might be involved in regulating CIS-induced cell apoptosis, and it might supply a new strategy to minimize CIS-induced nephrotoxicity.
	Kidney tissue		Days 1, 3, and 7.			
Liao et al./2017 [39]	Cells (HK-2)	RT-qPCR	42 h.	Upregulated: miR-140-5p	CIS exposure upregulated miR-140-5p in response to oxidative stress induced by CIS. It was also showed that MnSOD activity and cell vitality were increased, and LDH leakage was reduced in miR-140-5p overexpression. In fact, miR-140-5p directly targets the 3′-UTR of Nrf2 mRNA and increases the Nrf2 expression. The activation of Nrf2 pathway is a mechanism involved in ROS-protection by increased expression of antioxidant genes thus attenuating oxidative stress.	The overexpression of miR-140-5p after exposure to CIS may protect against CIS induced oxidative stress by activating Nrf2-dependent antioxidant pathway and provides a potentially therapeutic target in AKI.
	Kidney tissue		Days 1, 3, 5, 7, and 14.			
Yang et al./2019 [41]	Cells (HK-2)	RT-qPCR	24 h.	Upregulated: miR-199a-3p	MiR-199a-3p directly bound to mTOR 3′-untranslated region and reduced the expression and phosphorylation of mTOR. Moreover, p53 inhibited mTOR activation through activating miR-199a-3p. Blockade of miR-199a-3p significantly reduced CIS-induced cell apoptosis and inhibited caspase-3 activity.	P53 promoted miR-199a-3p expression both in vivo and in vitro, which subsequently inhibited mTOR signaling. So, it might provide a promising therapeutic target of AKI.
	Kidney tissue		Day 3.			
Yang et al./2019 [42]	Cells (HK-2)	RT-qPCR	24 h.	Downregulated: miR-26a	The upregulation of miR-26a (using miR- 26a mimics) alleviated the CIS-induced injury via the downregulation of TRPC6. Overexpression of miR-26a could attenuate CIS-induced cell injury. Upregulation of miR-26a could restrain Drp1 expression (an important mediator in regulating mitochondrial fission), which was consistent with the changes in TRPC6 expression. This means that the renoprotective effects of miR-26a against CIS-induced cells injury were inhibited through the mitochondrial apoptosis pathway.	MiR-26a can protect CIS-induced HK2 cell apoptosis via negatively regulating TRPC6 expression and may be targeted for the prevention and treatment of drug-related AKI.
	Kidney tissues		Day 3.			
Li et al./2021 [43]	Cells (HK-2)	RT-qPCR	24h.	Cells and kidney—Upregulated: miR-182-5p	Bioinformatic analysis predicted that miR-182-5p is a target gene for PRNCR1, and EZH1 was predicted to be a target gene for miR-182-5p. The study showed that miR-182-5p was negatively regulated by PRNCR1 and leed to upregulation of EZH1 expression. Overexpression of PRNCR1 attenuated CIS-induced apoptosis by downregulating the miR-182-5p/EZH1 axis.	The expression level of miR-182-5p was raised in mouse kidney and HK-2 cells after cisplatin treatment. miR-182-5p was the target gene of PRNCR1.
	Kidney		Days 1,3, and 7.			
Guo et al./2018 [40]	Cells (mPTC)	Microarray and qPCR	0, 2, 6, 12, and 24 h.	Upregulated: miR-709	The renal tubular mitochondrial dysfunction and cell apoptosis induced by cisplatin insult was almost completely blocked by anti–miR-709 management both in vitro and in vivo, suggesting a pathogenic role of miR-709 through mitochondrial damage in this kidney toxic injury model. MiR-709 targets the critical mitochondrial protective protein TFAM and impairs the biogenesis of the mitochondria in the renal tubular cells after acute insult.	Upregulation of renal tubular miR-709 after AKI mediates mitochondrial dysfunction and cell apoptosis by depressing TFAM expression. Targeting miR-709 may serve as a new approach to preserving mitochondrial function and preventing cell death in AKI.
	Kidney tissue		Day 3.			
Lee et al./2014 [35]	Cell (NRK-52E)	Microarray and RT-qPCR	24, 48, and 72 h.	Downregulated: miR-122 (the most downregulated) miR-10b miR-30e miR-193 miR-26a Upregulated: miR-34a (the most upregulated) let-7g	MiR-122 can be a direct suppressor of Foxo3 mRNA translation, while miR-34a activates Foxo3 by suppressing SIRT1. Increased expression and activation of Foxo3 has a role in triggering the p53 signaling pathway, culminating in cell apoptosis. Therefore, miR-122 and miR-34a dysregulation induces and actives Foxo3 contributing to CIS-induced acute tubular injury by fortifying the p53 signaling pathway.	The modulation of miR-122 and miR-34a could be a mechanism with which to prevent or treat AKI-induced by CIS.
	Kidney tissue		Days 1, 3, and 5.			
Xiong et al./2021 [44]	Cells (NRK-52E)	RT-qPCR	0, 12, 24, 36, 48 h	Cells and animals - Downregulated: miR-186	Overexpressing miR-186 could reverse the effects of cisplatin on NRK-52E cells proliferation and apoptosis. Moreover, inflammatory cytokines (IL-6, IL-1β, TNF-α, and Cox-2) expression was elevated by CIS; the increase of miR-186 reversed it, implying that increase of miR-186 repressed cell inflammatory response induced by CIS. ZEB1 was identified as miR-186 downstream target, which was found to be increased in AKI rat models. Knockdown of ZEB1 increased NRK-52E cell proliferation and restrained the apoptosis induced by CIS.	Loss of miR-186 expression contributed to CIS-induced AKI, partly through targeting ZEB1. MiR-186 might be provided an effective biomarker of AKI and a potential therapeutic target for its treatment.
	Serum and kidney		Days 0, 1, 3, and 5.			
Hao et al./2017 [38]	Cells (RPTC)	Microarray and RT-qPCR	0, 4, 8, 12, 16 h.	Kidney tissue-Microarray: both 1 and 3 days of CIS, FC ≥ 3 (all upregulated): miR-375 miR-503 miR-547 miR-212 miR-31* miR-743a	CIS treatment induces the activation of P53 and NF-κB, which collaboratively induce the expression of miR-375, which then represses the anti-apoptotic gene HNF-1β contributing to renal tubular cell injury and death (P53/NF-κB/miR-375/HNF-1β pathway in CIS-induced apoptosis).	It is suggested that miR-375 is an injurious miRNA. It may contribute to tubular cell injury and death during CIS nephrotoxicity. Delineation of P53/NF- κB/miR-375/HNF-1β pathway may provide novel therapeutic targets for kidney protection during CIS chemotherapy in cancer patients.
	Kidney tissues		Days 1 and 3.			
**Humans**						
**Author(s)/Year**	**Sample**	**Methods Used to Identify miRNAs**	**Time When the miRNAs Were Analyzed** **(after CIS Exposure/Administration)**	**miRNAs Differentially Expressed in Nephrotoxicity**	**Pathophysiological Implications of Dysregulated miRNAs** **(Related to the Authors’ Own Results)**	**Role of miRNAs as Biomarkers of Nephrotoxicity**
Quintanilha et al./2021 [45]	Plasma	NGS and RT-qPCR	Before and 5 days after.	Upregulated (before CIS administration in patients with grade ≥ 2 increased serum CRE): miR-3168 miR-4718 miR-6125	Bioinformatics analysis showed that upregulated miR-3168 targeting genes of the ErbB signaling pathway, which target PDK, could downregulate the pathway, leading to CIS-induced apoptosis in renal cells. The regulation of genes involved in the mitochondrial apoptosis pathway may also contribute to higher nephrotoxicity, suggested by a decrease in the activity of the anti-apoptotic protein Bcl-2 by miR-3168 and miR-6125. Genes of the CIS detoxification pathway, which includes the conjugation of CIS with glutathione, are also shown to be target of miR-3168 and miR-6125, which could reduce the content of glutathione S-transferase and reduced glutathione.	The evidence suggests the baseline plasmatic expression of miR-3168, miR-6125, and miR-4718 as potential predictors of CIS-induced nephrotoxicity, with miR-4718 being the most promising marker.
**Humans and Cells**						
**Author(s)/Year**	**Sample**	**Methods Used to Identify miRNAs**	**Time When the miRNAs Were Analyzed** **(after CIS Exposure/Administration)**	**miRNAs Differentially Expressed in Nephrotoxicity**	**Pathophysiological Implications of Dysregulated miRNAs** **(Related to the Authors’ Own Results)**	**Role of miRNAs as Biomarkers of Nephrotoxicity**
Pavkovic et al./2016 [46]	Cells (HPTEC)	qPCR	24 h.	Upregulated in human urine e downregulated in cells: miR-21 miR-200c miR-423 (Obs.: in humans, the miRNAs were high in patients with AKI diagnosis and also in patients without clinically proven AKI.)	Target prediction analysis of these miRNAs showed that the top pathway and associated pathological condition was found to be MYC-mediated apoptosis signaling and renal necrosis/cell death, respectively. In addition, they have several lapping targets including genes well-known in apoptosis as p21.	MiR-21, miR-200c, and miR-423 can be non-invasive and specific urinary biomarkers for the detection of drug-induced AKI in patients.
	Urine		Prior therapy and on subsequent time points: 4, 8, 12, 24, 48, 72, 96, 120, and 144 h.			

Abbreviations: AKI, acute kidney injury; BUN, blood urea nitrogen; CIS, cisplatin; CRE, creatinine; Drp1, dynamin-related protein 1; miRNA, microRNA; MLKL, mixed lineage kinase domain-like protein; mTOR, mechanistic target of rapamycin; NGS, next generation sequencing; NR, not reported; Nrf2, nuclear factor erythroid 2-related factor; PTEN, phosphatase and tensin homolog; ROS, reactive oxygen species; RT-qPCR, real-time quantitative polymerase chain reaction; TAK1, TGF-β-activated kinase; TGF-β, transforming growth factor-beta; TGFβR-1, TGF-β receptor 1; TRPC6, target transient receptor potential channel 6.

**Table 3:** List of miRNAs that were upregulated or downregulated in more than one study included in the systematic review.

miRNAs	Cells		Animals				Human		Total Number of Studies Where the miRNA Was Dysregulated
			Urine/Plasma		Kidney Tissue		Urine/Plasma		
	Upregulated	Downregulated	Upregulated	Downregulated	Upregulated	Downregulated	Upregulated	Downregulated	
miR-34a	Suter-Dick et al.,2018 [47]; Bhatt et al./2010 [34]; Lee at al./2014 [35]		Pavkovic et al., 2014 [52]; Wolenski et al./2017 [27]		Pavkovic et al., 2014 [52]; Wolenski et al./2017 [27]; El Magdoub et al., 2020 [30]; Bhatt et al./2010 [34]; Lee at al./2014 [35]				6
miR-21	Suter-Dick et al.,2018 [47]	Zhang et al., 2015 [25]; Pavkovic et al., 2016 [46]	Pavkovic et al., 2014 [52]; Okamoto et al./2021 [33]		Pavkovic et al., 2014 [52]		Pavkovic et al., 2016 [46]		5
let-7g-5p			Kanki et al., 2014 [51]; Wolenski et al., 2017 [27]	Kagawa et al., 2019 [29]	Lee at al./2014 [35]				4
miR-26a-5p		Yang et al., 2019 [42]	Okamoto et al./2021 [33]	Kagawa et al., 2019 [29]		Lee at al./2014 [35]; Yang et al., 2019 [42]			4
miR-192-5p	Suter-Dick et al./2018 [47]		Kanki et al., 2014 [51]; Pavkovic et al., 2014 [52]	Kagawa et al., 2019 [29]		Pavkovic et al., 2014 [52]			4
miR-30a-5p		Du et al., 2017 [37]	Kanki et al., 2014 [51]	Kagawa et al., 2019 [29]		Du et al., 2017 [37]			3
miR-15b-5p			Pavkovic et al., 2014 [52]; Glineur et al., 2018 [28]			Pavkovic et al., 2014 [52]			2
miR-20b-5p			Kanki et al., 2014 [51]; Pavkovic et al., 2014 [52]		Pavkovic et al., 2014 [52]				2
miR-25-3p			Kanki et al., 2014 [51]	Kagawa et al., 2019 [29]					2
miR-26b-5p			Wolenski et al., 2017 [27]			El Magdoub et al., 2020 [30]			2
miR-30e		Du et al., 2017 [37]				Lee at al./2014 [35]; Du et al., 2017 [37]			2
miR-34c-5p			Wolenski et al., 2017 [27]; Glineur et al., 2018 [28]						2
miR-122-5p		Lee at al./2014 [35]		Kagawa et al., 2019 [29]		Lee at al./2014 [35]			2
miR-130b-3p			Kanki et al., 2014 [51]; Wolenski et al., 2017 [27]						2
miR-140-3p			Kanki et al., 2014 [51]	Kagawa et al., 2019 [29]					2
miR-146a			Cho et al.,2017 [53]			Pavkovic et al., 2014 [52]			2
miR-181a	Zhu et al., 2012 [24]				Huang et al.,2020 [31]				2
miR-182-5p	Li et al., 2021 [43]		Glineur et al., 2018 [28]; Li et al., 2021 [43]						2
miR-183-5p			Kanki et al., 2014 [51]; Glineur et al., 2018 [28]						2
miR-191a-5p			Kanki et al., 2014 [51]	Kagawa et al., 2019 [29]					2
miR-193			Pavkovic et al., 2014 [52]			Pavkovic et al., 2014 [52]; Lee at al./2014 [35]			2
miR-210-3p			Pavkovic et al., 2014 [52]; Glineur et al., 2018 [28]			Pavkovic et al., 2014 [52]			2
miR-320-3p			Kanki et al., 2014 [51]; Harrill et al., 2017 [26]						2
miR-423-3p		Pavkovic et al., 2016 [46]	Glineur et al., 2018 [28]				Pavkovic et al., 2016 [46]		2
miR-500a-3p		Jiang et al.,2019 [48]; Zhang et al., 2020 [50]							2

## Data Availability

No new data were created or analyzed in this study. Data sharing is not applicable to this article.

## Supplementary Materials

The following are available online at https://www.mdpi.com/article/10.3390/ijms222312765/s1.
