# Supplementary material for: Dysregulated MicroRNAs as Biomarkers or Therapeutic Targets in Cisplatin-Induced Nephrotoxicity: A Systematic Review
Source: Int J Mol Sci. 2021 Nov 25;22(23):12765. doi: 10.3390/ijms222312765 (PMC8657822; doi:10.3390/ijms222312765)
Supplement: Supplementary file 1 [file ijms-22-12765-s001.zip › 1 - Supplementary Table S1. List of excluded studies.pdf]

Supplementary Table S1. List of excluded studies.

Reason for exclusion	Authors, year	Title	Reference
Comment	Seki, 2011	A commentary on microRNA-141 confers resistance to cisplatin-induced apoptosis by targeting YAP1 in human esophageal squamous cell carcinoma	[1]
	Akand et al./2020	Time to change the management of upper urinary tract urothelial carcinoma	[2]
Conference abstract	Joo et al./2010	miR-125b transcriptionally induced by Nrf2 inhibits AhR repressor for AhR activation	[3]
Correspondence	Wu et al./2017	Peri-operative chemotherapy with or without bevacizumab in operable oesophagogastric adenocarcinoma	[4]

Review	Nayeri and Babaknejad, 2016	Evaluation of novel biomarkers in nephrotoxicity	[5]
Kidney injury not developed by cisplatin	Pavkovic et al./2015	Glomerulonephritis-induced changes in urinary and kidney microRNA profiles in rats	[6]
Without quantification of miRNA or presentation of these data	Pellegrini et al./2014	MicroRNA-155 deficient mice experience heightened kidney toxicity when dosed with cisplatin	[7]
Assessment of exposure to cisplatin concomitantly with another drug	Joo et al./2013	miR-125b transcriptionally increased by Nrf2 inhibits AhR repressor, which protects kidney from cisplatin-induced injury	[8]
Without nephrotoxicity assessment	Pellegrini et al./2016	Application of small RNA sequencing to identify microRNAs in acute kidney injury and fibrosis	[9]

REFERENCES:

1. Seki N (2011) A commentary on microRNA-141 confers resistance to cisplatin-induced apoptosis by targeting YAP1 in human esophageal squamous

cell carcinoma. J Hum Genet 56:339–340. https://doi.org/10.1038/jhg.2011.26

2. Akand M, Muilwijk T, Aa F Van Der, Joniau S (2020) Time to change the management of upper urinary tract urothelial carcinoma. Cent Eur J Urol 73:381–382. https://doi.org/10.5173/ceju.2020.0181

3. Joo MS, Lee CG, Koo JH, Kim SG (2010) miR-125b transcriptionally induced by Nrf2 inhibits AhR repressor for AhR activation. J Pharm Pharmacol / Vol 56, Issue 3 Hum 0:3–5

4. Wu J, Qiu K, Zhu J (2017) Peri-operative chemotherapy with or without bevacizumab in operable oesophagogastric adenocarcinoma. Lancet Oncol 18:e241–e242. https://doi.org/10.1016/S1470-2045(17)30262-0

5. Nayeri H, Babaknejad N (2016) Evaluation of novel biomarkers in nephrotoxicity. Biomark Med 10:1209–1213. https://doi.org/10.2217/bmm-2016-0235

6. Pavkovic M, Riefke B, Frisk AL, et al (2015) Glomerulonephritis-induced changes in urinary and kidney microRNA profiles in rats. Toxicol Sci 145:348–359. https://doi.org/10.1093/toxsci/kfv053

7. Pellegrini KL, Han T, Bijol V, et al (2014) MicroRNA-155 deficient mice experience heightened kidney toxicity when dosed with cisplatin. Toxicol Sci 141:484–492. https://doi.org/10.1093/toxsci/kfu143

8. Joo MS, Lee CG, Koo JH, Kim SG (2013) miR-125b transcriptionally increased by Nrf2 inhibits AhR repressor, which protects kidney from cisplatin-induced injury. Cell Death Dis 4:e899-12. https://doi.org/10.1038/cddis.2013.427

9. Pellegrini KL, Gerlach C V., Craciun FL, et al (2016) Application of small RNA sequencing to identify microRNAs in acute kidney injury and fibrosis. Toxicol Appl Pharmacol 312:42–52. https://doi.org/10.1016/j.taap.2015.12.002
