# Supplementary material for: Dysregulated MicroRNAs as Biomarkers or Therapeutic Targets in Cisplatin-Induced Nephrotoxicity: A Systematic Review
Source: Int J Mol Sci. 2021 Nov 25;22(23):12765. doi: 10.3390/ijms222312765 (PMC8657822; doi:10.3390/ijms222312765)
Supplement: Supplementary file 1 [file ijms-22-12765-s001.zip › 2 - Supplementary table s2.pdf]

Supplementary table s2. Complete list of the 115 different miRNAs found differentially expressed in the studies included in this scoping review.

Table 1. MiRNAs upregulated and downregulated from the included studies in the scoping review.

**miRNAs**	**Cells**		**Animals**				**Human**		**Total number of studies where the miRNA was cited**
			**Urine/Plasma**		**Kidney tissue**		**Urine/ Plasma**		
	**Upregulated**	**Downregulated**	**Upregulated**	**Downregulated**	**Upregulated**	**Downregulated**	**Upregulated**	**Downregulate d**	
**miR-34a**	Suter-Dick et al.,2018 [20]; Bhatt et al./2010 [35]; Lee at al./2014 [36]		Pavkovic et al., 2014 [25]; Wolenski et al./2017 [28]		Pavkovic et al., 2014 [25]; Wolenski et al./2017 [28]; El Magdoub et al., 2020 [31]; Bhatt et al./2010 [35]; Lee at al./2014 [36]				6
**miR-21**	Suter-Dick et al.,2018 [20]	Zhang et al., 2015 [18]; Pavkovic et al., 2016 [46]	Pavkovic et al., 2014 [25]; Okamoto et al./2021 [34]		Pavkovic et al., 2014 [25]		Pavkovic et al., 2016 [46]		5
**let-7g-5p**			Kanki et al., 2014 [24]; Wolenski et al., 2017 [28]	Kagawa et al., 2019 [30]	Lee at al./2014 [36]				4
**miR-26a-5p**		Yang et al., 2019 [42]	Okamoto et al./2021 [34]	Kagawa et al., 2019 [30]		Lee at al./2014 [36]; Yang et al., 2019 [42]			4
**miR-192-5p**	Suter-Dick et al./2018 [20]		Kanki et al., 2014 [24]; Pavkovic et al., 2014 [25]	Kagawa et al., 2019 [30]		Pavkovic et al., 2014 [25]			4
**miR-30a-5p**		Du et al., 2017 [37]	Kanki et al., 2014 [24]	Kagawa et al., 2019 [30]		Du et al., 2017 [37]			3
**miR-15b-5p**			Pavkovic et al., 2014 [25];			Pavkovic et al., 2014 [25]			2

			Glineur et al., 2018 [29]				
**miR-20b-5p**			Kanki et al., 2014 [24]; Pavkovic et al., 2014 [25]		Pavkovic et al., 2014 [25]		2
**miR-25-3p**			Kanki et al., 2014 [24]	Kagawa et al., 2019 [30]			2
**miR-26b-3p**			Kanki et al., 2014 [24]				1
**miR-26b-5p**			Wolenski et al., 2017 [28]			El Magdoub et al., 2020 [31]	2
**miR-30e**		Du et al., 2017 [37]				Lee at al./2014 [36]; Du et al., 2017 [37]	2
**miR-34c-5p**			Wolenski et al., 2017 [28]; Glineur et al., 2018 [29]				2
**miR-122-5p**		Lee at al./2014 [36]		Kagawa et al., 2019 [30]		Lee at al./2014 [36]	2
**miR-130b-3p**			Kanki et al., 2014 [24]; Wolenski et al., 2017 [28]				2
**miR-140-3p**			Kanki et al., 2014 [24]	Kagawa et al., 2019 [30]			2
**miR-140-5p**			Wolenski et al., 2017 [28]				1
**miR-146a**			Cho et al.,2017 [26]			Pavkovic et al., 2014 [25]	2
**miR-181a**	Zhu et al., 2012 [17]				Huang et al.,2020 [32]		2
**miR-182-5p**	Li et al., 2021 [43]		Glineur et al., 2018 [29]; Li et al., 2021 [43]				2

**miR-183-5p**			Kanki et al., 2014 [24]; Glineur et al., 2018 [29]						2
**miR-191a-5p**			Kanki et al., 2014 [24]	Kagawa et al., 2019 [30]					2
**miR-193**			Pavkovic et al., 2014 [25]			Pavkovic et al., 2014 [25]; Lee at al./2014 [36]			2
**miR-210-3p**			Pavkovic et al., 2014 [25]; Glineur et al., 2018 [29]			Pavkovic et al., 2014 [25]			2
**miR-320-3p**			Kanki et al., 2014 [24]; Harrill et al., 2017 [27]						2
**miR-423-3p**		Pavkovic et al., 2016 [46]	Glineur et al., 2018 [29]				Pavkovic et al., 2016 [46]		2
**miR-500a-3p**		Jiang et al.,2019 [21]; Zhang et al.,2020 [23]							2
**let-7a-1-3p**			Kanki et al., 2014 [24]						1
**let-7b**						El Magdoub et al., 2020 [31]			1
**let-7c-5p**				Kagawa et al., 2019 [30]					1
**let-7f-5p**				Kagawa et al., 2019 [30]					1
**let-7i-5p**				Kagawa et al., 2019 [30]					1
**miR-7a-1-3p**			Kanki et al., 2014 [24]						1
**miR-9-3p**	Wu et al.,2019 [22]								1

**miR-10a**			Okamoto et al./2021 [34]				1
**miR-10b**						Lee at al./2014 [36]	1
**miR-15**			Pavkovic et al., 2014 [25]				1
**miR-16**			Pavkovic et al., 2014 [25]			Pavkovic et al., 2014 [25]	1
**miR-17-5p**			Kanki et al., 2014 [24]				1
**miR-20a**			Pavkovic et al., 2014 [25]			Pavkovic et al., 2014 [25]	1
**miR-23a-3p**				Kagawa et al., 2019 [30]			1
**miR-23b-5p**		Wu et al.,2019 [22]					1
**miR-27b-5p**		Wu et al.,2019 [22]					1
**miR-29**		Zhang et al., 2015 [18]					1
**miR-29a**	Suter-Dick et al.,2018 [20]						1
**miR-30b**		Du et al., 2017 [37]				Du et al., 2017 [37]	1
**miR-30c**		Du et al., 2017 [37]				Du et al., 2017 [37]	1
**miR-30d**		Du et al., 2017 [37]				Du et al., 2017 [37]	1
**miR-31-3p**					Hao et al., 2017 [38]		1
**miR-31-5p**					Wu et al., 2020 [33]		1
**miR-93-5p**			Kanki et al., 2014 [24]				1
**miR-128**			Wolenski et al., 2017 [28]				1

**miR-130a**			Harrill et al., 2017 [27]				1
**miR-138**			Harrill et al., 2017 [27]				1
**miR-140-5p**	Liao et al., 2017 [39]				Liao et al., 2017 [39]		1
**miR-141**						Pavkovic et al., 2014 [25]	1
**miR-143-3p**				Kagawa et al., 2019 [30]			1
**miR-146b-5p**				Kagawa et al., 2019 [30]			1
**miR-148a-3p**				Kagawa et al., 2019 [30]			1
**miR-151-3p**			Harrill et al., 2017 [27]				1
**miR-152**			Harrill et al., 2017 [27]				1
**miR-155-5p**			Glineur et al., 2018 [29]				1
**miR-184**					Pavkovic et al., 2014 [25]		1
**miR-185**			Pavkovic et al., 2014 [25]			Pavkovic et al., 2014 [25]	1
**miR-186**		Xiong et al., 2021 [44]		Xiong et al., 2021 [44]		Xiong et al., 2021 [44]	1
**miR-193-5p**			Kanki et al., 2014 [24]				1
**miR-193a-3p**			Glineur et al., 2018 [29]				1
**miR-194-5p**	Jiang et al.,2019 [21]						1
**miR-196c**						Pavkovic et al., 2014 [25]	1
**miR-199a-3p**	Yang et al., 2019 [41]				Yang et al., 2019 [41]		1

**miR-200b**			Pavkovic et al., 2014 [25]			Pavkovic et al., 2014 [25]		1
**miR-200c**		Pavkovic et al., 2016 [46]					Pavkovic et al., 2016 [46]	1
**miR-205**		Zhang et al., 2015 [18]						1
**miR-212**					Hao et al., 2017 [38]			1
**miR-215**				Kagawa et al., 2019 [30]				1
**miR-218**			Harrill et al., 2017 [27]					1
**miR-218a-5p**			Kanki et al., 2014 [24]					1
**miR-221**			Harrill et al., 2017 [27]					1
**miR-223**						Pavkovic et al., 2014 [25]		1
**miR-297**	Wu et al.,2019 [22]							1
**miR-327**					Pavkovic et al., 2014 [25]			1
**miR-328**			Harrill et al., 2017 [27]					1
**miR-328a-3p**			Kanki et al., 2014 [24]					1
**miR-335**			Kanki et al., 2014 [24]					1
**miR-339-3p**			Pavkovic et al., 2014 [25]					1
**miR-340-5p**			Kanki et al., 2014 [24]					1
**miR-371b-5p**	Wu et al.,2019 [22]							1
**miR-375**	Hao et al., 2017 [38]				Hao et al., 2017 [38]			1

**miR-378a**			Wolenski et al., 2017 [28]			1
**miR-378a-3p**				Kagawa et al., 2019 [30]		1
**miR-378a-5p**			Kanki et al., 2014 [24]			1
**miR-449**	Qin et al.,2016 [19]					1
**miR-486**				Kagawa et al., 2019 [30]		1
**miR-503**					Hao et al., 2017 [38]	1
**miR-532-3p**			Kanki et al., 2014 [24]			1
**miR-547**					Hao et al., 2017 [38]	1
**miR-577**	Jiang et al.,2019 [21]					1
**miR-680**			Harrill et al., 2017 [27]			1
**miR-685**			Harrill et al., 2017 [27]			1
**miR-709**	Guo et al., 2018 [40]				Guo et al., 2018 [40]	1
**miR-743a**					Hao et al., 2017 [38]	1
**miR-744-5p**			Kanki et al., 2014 [24]			1
**miR-1236-5p**		Wu et al.,2019 [22]				1
**miR-1839**			Wolenski et al., 2017 [28]			1
**miR-1839-5p**			Kanki et al., 2014 [24]			1
**miR-3135b**	Wu et al.,2019 [22]					1

**miR-3168**						Quintanilha et al., 2021 [45]	1
**miR-3605-5p**		Wu et al.,2019 [22]					1
**miR-4299**	Wu et al.,2019 [22]						1
**miR-4440**	Wu et al.,2019 [22]						1
**miR-4485**	Wu et al.,2019 [22]						1
**miR-4718**						Quintanilha et al., 2021 [45]	1
**miR-4793-3p**	Wu et al.,2019 [22]						1
**miR-6125**						Quintanilha et al., 2021 [45]	1
**miR-6841-5p**		Wu et al.,2019 [22]					1

References

1. Jelinek, M.J.; Lee, S.M.; Wyche Okpareke, A.; Wing, C.; Koyner, J.L.; Murray, P.T.; Stadler, W.M.; O' Donnell, P.H. Predicting Acute Renal Injury in Cancer Patients Receiving Cisplatin Using Urinary Neutrophil Gelatinase-Associated Lipocalin and Cystatin C. *Clin. Transl. Sci.* **2018**, *11*, 420–427, doi:10.1111/cts.12547.

2. Pabla, N.; Dong, Z. Cisplatin nephrotoxicity: Mechanisms and renoprotective strategies. *Kidney Int.* **2008**, *73*, 994–1007, doi:10.1038/sj.ki.5002786.

3. Waikar, S.S.; Betensky, R.A.; Emerson, S.C.; Bonventre, J. V. Imperfect gold standards for kidney injury biomarker evaluation. *J. Am. Soc. Nephrol.* **2012**, *23*, 13–21, doi:10.1681/ASN.2010111124.

4. Van Meer, L.; Moerland, M.; Cohen, A.F.; Burggraaf, J. Urinary kidney biomarkers for early detection of nephrotoxicity in clinical drug development. *Br. J. Clin. Pharmacol.* **2014**, *77*, 947–957, doi:10.1111/bcp.12282.

5. Waikar, S.S.; Sabbisetti, V.S.; Bonventre, J. V. Normalization of urinary biomarkers to creatinine during changes in glomerular filtration rate. *Kidney Int.* **2010**, *78*, 486–494, doi:10.1038/ki.2010.165.

6. Saliminejad, K.; Khorram Khorshid, H.R.; Soleymani Fard, S.; Ghaffari, S.H. An overview of microRNAs: Biology, functions, therapeutics, and analysis methods. *J. Cell. Physiol.* **2019**, *234*, 5451–5465, doi:10.1002/jcp.27486.

7. Krol, J.; Loedige, I.; Filipowicz, W. The widespread regulation of microRNA biogenesis, function and decay. *Nat. Rev. Genet.* **2010**, *11*, 597–610, doi:10.1038/nrg2843.

8. Zhang, W.; Duan, N.; Zhang, Q.; Song, T.; Li, Z.; Zhang, C.; Chen, X.; Wang, K. DNA methylation mediated down-regulation of miR-370 regulates cell growth through activation of the Wnt/β-catenin signaling pathway in human osteosarcoma cells. *Int. J. Biol. Sci.* **2017**, *13*, 561–573, doi:10.7150/ijbs.19032.

9. Ambros, V. MicroRNAs and Developmental Timing. *Curr Opin Genet Dev* **2011**, *21*, 511–517, doi:10.1016/j.gde.2011.04.003.MicroRNAs.

10. Small, E.M.; Olson, E.N. Pervasive roles of microRNAs in cardiovascular biology. *Nature* **2011**, *469*, 336–342, doi:10.1038/nature09783.

11. Chong, M.M.W.; Zhang, G.; Cheloufi, S.; Neubert, T.A.; Hannon, G.J.; Littman, D.R. Canonical and alternate functions of the microRNA biogenesis machinery. *Genes Dev.* **2010**, *24*, 1951–1960, doi:10.1101/gad.1953310.

12. Li, Y.F.; Jing, Y.; Hao, J.; Frankfort, N.C.; Zhou, X.; Shen, B.; Liu, X.; Wang, L.; Li, R. MicroRNA-21 in the pathogenesis of acute kidney injury. *Protein Cell* **2013**, *4*, 813–819, doi:10.1007/s13238-013-3085-y.

13. Weber, J.A.; Baxter, D.H.; Zhang, S.; Huang, D.Y.; Huang, K.H.; Lee, M.J.; Galas, D.J.; Wang, K. The microRNA spectrum in 12 body fluids. *Clin. Chem.* **2010**, *56*, 1733–1741, doi:10.1373/clinchem.2010.147405.

14. Koturbash, I.; Tolleson, W.H.; Guo, L.; Yu, D.; Chen, S.; Hong, H.; Mattes, W.; Ning, B. MicroRNAs as pharmacogenomic biomarkers for drug efficacy and drug safety assessment. *Biomark. Med.* **2015**, *9*, 1153–1176, doi:10.2217/bmm.15.89.

15. Roderburg, C.; Luedde, T. Circulating microRNAs as markers of liver inflammation, fibrosis and cancer. *J. Hepatol.* **2014**, *61*, 1434–1437, doi:10.1016/j.jhep.2014.07.017.

16. Rupaimoole, R.; Slack, F.J. MicroRNA therapeutics: Towards a new era for the management of cancer and other diseases. *Nat. Rev. Drug Discov.* **2017**, *16*, 203–221, doi:10.1038/nrd.2016.246.

17. Zhu, H.Y.; Liu, M.Y.; Hong, Q.; Zhang, D.; Geng, W.J.; Xie, Y.S.; Chen, X.M. Role of microRNA-181a in the apoptosis of tubular epithelial cell induced by cisplatin. *Chin. Med. J. (Engl).* **2012**, *125*, 523–526, doi:10.3760/cma.j.issn.0366-6999.2012.03.022.

18. Zhang, H.; Zhang, X.; Yuan, X.; Wang, L.; Xiao, Y. MicroRNA-205 inhibits renal cells apoptosis via targeting CMTM4. *Iran. J. Basic Med. Sci.* **2015**, *18*, 1020–1026, doi:10.22038/ijbms.2015.5467.

19. Qin, W.; Xie, W.; Yang, X.; Xia, N.; Yang, K. Inhibiting microrna-449 attenuates cisplatin-induced injury in nrk-52e cells possibly via regulating the sirt1/p53/bax pathway. *Med. Sci. Monit.* **2016**, *22*, 818–823, doi:10.12659/MSM.897187.

20. Suter-Dick, L.; Mauch, L.; Ramp, D.; Caj, M.; Vormann, M.K.; Hutter, S.; Lanz, H.L.; Vriend, J.; Masereeuw, R.; Wilmer, M.J. Combining Extracellular miRNA Determination with Microfluidic 3D Cell Cultures for the Assessment of Nephrotoxicity: a Proof of Concept Study. *AAPS J.* **2018**, *20*, 1–9, doi:10.1208/s12248-018-0245-2.

21. Jiang, L.; Liu, X.Q.; Ma, Q.; Yang, Q.; Gao, L.; Li, H. Di; Wang, J.N.; Wei, B.; Wen, J.; Li, J.; et al. hsa-miR-500a-3P alleviates kidney injury by targeting MLKL-mediated necroptosis in renal epithelial cells. *FASEB J.* **2019**, *33*, 3523–3535, doi:10.1096/fj.201801711R.

22. Wu, J.; Li, D. dan; Li, J. yao; Yin, Y. cong; Li, P. chang; Qiu, L.; Chen, L. meng Identification of microRNA-mRNA networks involved in cisplatin-induced renal tubular epithelial cells injury. *Eur. J. Pharmacol.* **2019**, *851*, 1–12, doi:10.1016/j.ejphar.2019.02.015.

23. Zhang, S.; Sun, H.; Kong, W.; Zhang, B. Functional role of microRNA-500a-3P-loaded liposomes in the treatment of cisplatininduced AKI. *IET Nanobiotechnology* **2020**, *14*, 465–469, doi:10.1049/iet-nbt.2019.0247.

24. Kanki, M.; Moriguchi, A.; Sasaki, D.; Mitori, H.; Yamada, A.; Unami, A.; Miyamae, Y. Identification of urinary miRNA biomarkers for detecting cisplatin-induced proximal tubular injury in rats. *Toxicology* **2014**, *324*, 158–168, doi:10.1016/j.tox.2014.05.004.

25. Pavkovic, M.; Riefke, B.; Ellinger-Ziegelbauer, H. Urinary microRNA profiling for identification of biomarkers after cisplatin-induced kidney injury. *Toxicology* **2014**, *324*, 147–157, doi:10.1016/j.tox.2014.05.005.

26. Cho, Y.E.; Kim, S.H.; Lee, B.H.; Baek, M.C. Circulating plasma and exosomal microRNAs as indicators of drug-induced organ injury in rodent models. *Biomol. Ther.* **2017**, *25*, 367–373, doi:10.4062/biomolther.2016.174.

27. Harrill, A.H.; Lin, H.; Tobacyk, J.; Seely, J.C. Mouse population-based evaluation of urinary protein and miRNA biomarker performance associated with cisplatin renal injury. *Exp. Biol. Med.* **2017**, *243*, 1–11, doi:10.1177/1535370217740854.

28. Wolenski, F.S.; Shah, P.; Sano, T.; Shinozawa, T.; Bernard, H.; Gallacher, M.J.; Wyllie, S.D.; Varrone, G.; Cicia,

L.A.; Carsillo, M.E.; et al. Identification of microRNA biomarker candidates in urine and plasma from rats with kidney or liver damage. *J. Appl. Toxicol.* **2017**, *37*, 278–286, doi:10.1002/jat.3358.

29. Glineur, S.F.; Hanon, E.; Dremier, S.; Snelling, S.; Berteau, C.; De Ron, P.; Nogueira da Costa, A. Assessment of a Urinary Kidney MicroRNA Panel as Potential Nephron Segment-Specific Biomarkers of Subacute Renal Toxicity in Preclinical Rat Models. *Toxicol. Sci.* **2018**, *166*, 409–419, doi:10.1093/toxsci/kfy213.

30. Kagawa, T.; Zárybnický, T.; Omi, T.; Shirai, Y.; Toyokuni, S.; Oda, S.; Yokoi, T. A scrutiny of circulating microRNA biomarkers for drug-induced tubular and glomerular injury in rats. *Toxicology* **2019**, *415*, 26–36, doi:10.1016/j.tox.2019.01.011.

31. El Magdoub, H.M.; Schaalan, M.F.; Rahmo, R.M.; Farag, D.B.; Khedr, L.H. Implications of miRNAs on TGF-β/TAK1/mTOR pathway in mediating the renoprotective effects of pentoxifylline against cisplatin-induced nephrotoxicity in rats. *Toxicol. Appl. Pharmacol.* **2020**, *404*, 115184, doi:10.1016/j.taap.2020.115184.

32. Huang, S.J.; Huang, J.; Yan, Y.B.; Qiu, J.; Tan, R.Q.; Liu, Y.; Tian, Q.; Guan, L.; Niu, S.S.; Zhang, Y.; et al. The renoprotective effect of curcumin against cisplatin-induced acute kidney injury in mice: involvement of miR-181a/PTEN axis. *Ren. Fail.* **2020**, *42*, 350–357, doi:10.1080/0886022X.2020.1751658.

33. Wu, Z.; Li, C.; Li, Q.; Li, J.; Lu, X. Puerarin alleviates cisplatin-induced acute renal damage and upregulates microRNA-31-related signaling. *Exp. Ther. Med.* **2020**, *20*, 3122–3129, doi:10.3892/etm.2020.9081.

34. Okamoto, M.; Ichii, O.; Mie, K.; Nishida, H.; Akiyoshi, H. Altered clinicopathology and renal pathology in dogs treated with a clinical dose of cisplatin. *Jpn. J. Vet. Res.* **2021**, *69*, 109–124, doi:10.14943/jjvr.69.2.109.

35. Bhatt, K.; Zhou, L.; Mi, Q.S.; Huang, S.; She, J.X.; Dong, Z. MicroRNA-34a is induced via p53 during cisplatin nephrotoxicity and contributes to cell survival. *Mol. Med.* **2010**, *16*, 409–416, doi:10.2119/molmed.2010.00002.

36. Lee, C.G.; Kim, J.G.; Kim, H.J.; Kwon, H.K.; Cho, I.J.; Choi, D.W.; Lee, W.H.; Kim, W.D.; Hwang, S.J.; Choi, S.; et al. Discovery of an integrative network of microRNAs and transcriptomics changes for acute kidney injury. *Kidney Int.* **2014**, *86*, 943–953, doi:10.1038/ki.2014.117.

37. Du, B.; Dai, X.M.; Li, S.; Qi, G.L.; Cao, G.X.; Zhong, Y.; Yin, P. di; Yang, X.S. MiR-30c regulates cisplatin-induced apoptosis of renal tubularc epithelial cells by targeting Bnip3L and Hspa5. *Cell Death Dis.* **2017**, *8*, 1–13, doi:10.1038/cddis.2017.377.

38. Hao, J.; Lou, Q.; Wei, Q.; Mei, S.; Li, L.; Wu, G.; Mi, Q.S.; Mei, C.; Dong, Z. MicroRNA-375 is induced in cisplatin nephrotoxicity to repress hepatocyte nuclear factor 1-β. *J. Biol. Chem.* **2017**, *292*, 4571–4582, doi:10.1074/jbc.M116.754929.

39. Liao, W.; Fu, Z.; Zou, Y.; Wen, D.; Ma, H.; Zhou, F.; Chen, Y.; Zhang, M.; Zhang, W. MicroRNA-140-5p attenuated oxidative stress in Cisplatin induced acute kidney injury by activating Nrf2/ARE pathway through a Keap1-independent mechanism. *Exp. Cell Res.* **2017**, *360*, 292–302, doi:10.1016/j.yexcr.2017.09.019.

40. Guo, Y.; Ni, J.; Chen, S.; Bai, M.; Lin, J.; Ding, G.; Zhang, Y.; Sun, P.; Jia, Z.; Huang, S.; et al. MicroRNA-709 Mediates Acute Tubular Injury through Effects on Mitochondrial Function. *J. Am. Soc. Nephrol.* **2018**, *29*, 449–461,

doi:10.1681/ASN.2017040381.

41. Yang, A.; Liu, F.; Guan, B.; Luo, Z.; Lin, J.; Fang, W.; Liu, L.; Zuo, W. p53 induces miR-199a-3p to suppress mechanistic target of rapamycin activation in cisplatin-induced acute kidney injury. *J. Cell. Biochem.* **2019**, *120*, 17625–17634, doi:10.1002/jcb.29030.

42. Yang, Y.; Li, Z.L.; Wang, F.M.; Tang, R.N.; Tu, Y.; Liu, H. MicroRNA26a inhibits cisplatin-induced renal tubular epithelial cells apoptosis through suppressing the expression of transient receptor potential channel 6 mediated dynamin-related protein 1. *Cell Biochem. Funct.* **2019**, *38*, 384–391, doi:10.1002/cbf.3474.

43. Li, J.; Fan, X.; Wang, Q.; Gong, Y.; Guo, L. Long noncoding RNA PRNCR1 reduces renal epithelial cell apoptosis in cisplatin-induced AKI by regulating miR-182-5p/EZH1. *Kidney Blood Press. Res.* **2021**, *46*, 162–172, doi:10.1159/000510157.

44. Xiong, X.; Tang, B.; Ji, T.; Li, X.; Bai, S. Ameliorative effects of miR-186 on cisplatin-Triggered acute kidney injury via targeting ZEB1. *Am. J. Transl. Res.* **2021**, *13*, 4296–4308.

45. Quintanilha, J.C.F.; Cursino, M.A.; Borges, J.B.; Torso, N.G.; Bastos, L.B.; Oliveira, J.M.; Cobaxo, T.S.; Pincinato, E.C.; Hirata, M.H.; Geraldo, M. V; et al. MiR-3168, miR-6125, and miR-4718 as potential predictors of cisplatin-induced nephrotoxicity in patients with head and neck cancer. *BMC Cancer* **2021**, *21*, 1–10.

46. Pavkovic, M.; Robinson-Cohen, C.; Chua, A.S.; Nicoara, O.; Cárdenas-González, M.; Bijol, V.; Ramachandran, K.; Hampson, L.; Pirmohamed, M.; Antoine, D.J.; et al. Detection of drug-induced acute kidney injury in humans using urinary KIM-1, miR-21, -200c, and -423. *Toxicol. Sci.* **2016**, *152*, 205–213, doi:10.1093/toxsci/kfw077.

47. Mehta, R.L.; Kellum, J.A.; Shah, S. V.; Molitoris, B.A.; Ronco, C.; Warnock, D.G.; Levin, A.; Bagga, A.; Bakkaloglu, A.; Bonventre, J. V.; et al. Acute kidney injury network: Report of an initiative to improve outcomes in acute kidney injury. *Crit. Care* **2007**, *11*, 1–8, doi:10.1186/cc5713.

48. Arroyo, J.D.; Chevillet, J.R.; Kroh, E.M.; Ruf, I.K.; Pritchard, C.C.; Gibson, D.F.; Mitchell, P.S.; Bennett, C.F.; Pogosova-Agadjanyan, E.L.; Stirewalt, D.L.; et al. Argonaute2 complexes carry a population of circulating microRNAs independent of vesicles in human plasma. *Proc. Natl. Acad. Sci. U. S. A.* **2011**, *108*, 5003–5008, doi:10.1073/pnas.1019055108.

49. Cortez, M.A.; Calin, G.A. MicroRNA identification in plasma and serum: A new tool to diagnose and monitor diseases. *Expert Opin. Biol. Ther.* **2009**, *9*, 703–711, doi:10.1517/14712590902932889.

50. Melkonyan, H.S.; Feaver, W.J.; Meyer, E.; Scheinker, V.; Shekhtman, E.M.; Xin, Z.; Umansky, S.R. Transrenal nucleic acids: From proof of principle to clinical tests. *Ann. N. Y. Acad. Sci.* **2008**, *1137*, 73–81, doi:10.1196/annals.1448.015.

51. Hermeking, H. The miR-34 family in cancer and apoptosis. *Cell Death Differ.* **2010**, *17*, 193–199, doi:10.1038/cdd.2009.56.

52. Chang, T.C.; Wentzel, E.A.; Kent, O.A.; Ramachandran, K.; Mullendore, M.; Lee, K.H.; Feldmann, G.; Yamakuchi, M.; Ferlito, M.; Lowenstein, C.J.; et al. Transactivation of miR-34a by p53 Broadly Influences Gene Expression

and Promotes Apoptosis. *Mol. Cell* **2007**, *26*, 745–752, doi:10.1016/j.molcel.2007.05.010.

53. Manohar, S.; Leung, N. Cisplatin nephrotoxicity: a review of the literature. *J. Nephrol.* **2018**, *31*, 15–25, doi:10.1007/s40620-017-0392-z.

54. Ong, A.L.C.; Ramasamy, T.S. Role of Sirtuin1-p53 regulatory axis in aging, cancer and cellular reprogramming. *Ageing Res. Rev.* **2018**, *43*, 64–80, doi:10.1016/J.ARR.2018.02.004.

55. Zhu, S.; Pabla, N.; Tang, C.; He, L.; Dong, Z. DNA damage response in cisplatin-induced nephrotoxicity. *Arch. Toxicol.* **2015**, *89*, 2197–2205, doi:10.1007/s00204-015-1633-3.

56. Chung, A.C.K.; Yu, X.; Lan, H.Y. MicroRNA and nephropathy: Emerging concepts. *Int. J. Nephrol. Renovasc. Dis.* **2013**, *6*, 169–179, doi:10.2147/IJNRD.S37885.

57. Endale, M.; Kim, T.H.; Kwak, Y.S.; Kim, N.M.; Kim, S.H.; Cho, J.Y.; Yun, B.S.; Rhee, M.H. Torilin Inhibits Inflammation by Limiting TAK1-Mediated MAP Kinase and NF- B Activation. *Mediators Inflamm.* **2017**, *2017*, 1–13, doi:10.1155/2017/7250968.

58. Saal, S.; Harvey, S.J. MicroRNAs and the kidney: coming of age. *Curr. Opin. Nephrol. Hypertens.* **2009**, *18*, 317–323, doi:10.1097/MNH.0b013e32832c9da2.

59. Ma, L.; Qu, L. The function of MicroRNAs in renal development and pathophysiology. *J. Genet. Genomics* **2013**, *40*, 143–152, doi:10.1016/j.jgg.2013.03.002.

60. Du, J.; Cao, X.; Zou, L.; Chen, Y.; Guo, J.; Chen, Z.; Hu, S.; Zheng, Z. MicroRNA-21 and Risk of Severe Acute Kidney Injury and Poor Outcomes after Adult Cardiac Surgery. *PLoS One* **2013**, *8*, 1–8, doi:10.1371/journal.pone.0063390.

61. Zununi Vahed, S.; Omidi, Y.; Ardalan, M.; Samadi, N. Dysregulation of urinary miR-21 and miR-200b associated with interstitial fibrosis and tubular atrophy (IFTA) in renal transplant recipients. *Clin. Biochem.* **2017**, *50*, 32–39, doi:10.1016/j.clinbiochem.2016.08.007.

62. Zhong, X.; Chung, A.C.K.; Chen, H.Y.; Dong, Y.; Meng, X.M.; Li, R.; Yang, W.; Hou, F.F.; Lan, H.Y. MiR-21 is a key therapeutic target for renal injury in a mouse model of type 2 diabetes. *Diabetologia* **2013**, *56*, 663–674, doi:10.1007/s00125-012-2804-x.

63. Thum, T.; Catalucci, D.; Bauersachs, J. MicroRNAs: novel regulators in cardiac development and disease. *Cardiovasc. Res.* **2008**, *79*, 562–570, doi:10.1093/cvr/cvn137.

64. Godwin, J.G.; Ge, X.; Stephan, K.; Jurisch, A.; Tullius, S.G.; Iacomini, J. Identification of a microRNA signature of renal ischemia reperfusion injury. *Proc. Natl. Acad. Sci. U. S. A.* **2010**, *107*, 14339–14344, doi:10.1073/pnas.0912701107.

65. Liu, G.; Friggeri, A.; Yang, Y.; Milosevic, J.; Ding, Q.; Thannickal, V.J.; Kaminski, N.; Abraham, E. miR-21 mediates fibrogenic activation of pulmonary fibroblasts and lung fibrosis. *J. Exp. Med.* **2010**, *207*, 1589–1597, doi:10.1084/jem.20100035.

66. Seo, J.; Nam, Y.W.; Kim, S.; Oh, D.B.; Song, J. Necroptosis molecular mechanisms: Recent findings regarding novel necroptosis regulators. *Exp. Mol. Med.* **2021**, *53*, 1007–1017, doi:10.1038/s12276-021-00634-7.

67. Cai, Z.; Jitkaew, S.; Zhao, J.; Chiang, H.C.; Choksi, S.; Liu, J.; Ward, Y.; Wu, L.G.; Liu, Z.G. Plasma membrane translocation of trimerized MLKL protein is required for TNF-induced necroptosis. *Nat. Cell Biol.* **2014**, *16*, 55–65, doi:10.1038/ncb2883.

68. Priante, G.; Gianesello, L.; Ceol, M.; Del Prete, D.; Anglani, F. Cell death in the kidney. *Int. J. Mol. Sci.* **2019**, *20*, 1–21, doi:10.3390/ijms20143598.

69. Linkermann, A.; Bräsen, J.H.; Darding, M.; Jin, M.K.; Sanz, A.B.; Heller, J.O.; De Zen, F.; Weinlich, R.; Ortiz, A.; Walczak, H.; et al. Two independent pathways of regulated necrosis mediate ischemia-reperfusion injury. *Proc. Natl. Acad. Sci. U. S. A.* **2013**, *110*, 12024–12029, doi:10.1073/pnas.1305538110.

70. Li, Z.; Rana, T.M. Therapeutic targeting of microRNAs: current status and future challenges. *Nat. Rev. Drug Discov.* 2014, *13*, 622–638.

71. Beg, M.S.; Brenner, A.J.; Sachdev, J.; Borad, M.; Kang, Y.K.; Stoudemire, J.; Smith, S.; Bader, A.G.; Kim, S.; Hong, D.S. Phase I study of MRX34, a liposomal miR-34a mimic, administered twice weekly in patients with advanced solid tumors. *Invest. New Drugs* **2017**, *35*, 180–188, doi:10.1007/s10637-016-0407-y.

72. Tricco, A.C.; Lillie, E.; Zarin, W.; Al, E. PRISMA Extension for Scoping Reviews (PRISMA-ScR): Checklist and Explanation. *Ann. Intern. Med.* **2018**, *169*, 467–473.
