# Supplementary material for: Dysregulated MicroRNAs as Biomarkers or Therapeutic Targets in Cisplatin-Induced Nephrotoxicity: A Systematic Review
Source: Int J Mol Sci. 2021 Nov 25;22(23):12765. doi: 10.3390/ijms222312765 (PMC8657822; doi:10.3390/ijms222312765)
Supplement: Supplementary file 1 [file ijms-22-12765-s001.zip › 3 - Supplementary file 1. Search Strategy.pdf]

Supplementary file 1. Search strategies of the scoping review

A. PUBMED

((((MicroRNAs[MeSH Terms]) OR (MicroRNAs[Title/Abstract] OR "MicroRNA"[Title/Abstract] OR miRNAs[Title/Abstract] OR "Micro RNA"[Title/Abstract] OR "RNA, Micro"[Title/Abstract] OR miRNA[Title/Abstract] OR "Primary MicroRNA"[Title/Abstract] OR "MicroRNA, Primary"[Title/Abstract] OR "Primary miRNA"[Title/Abstract] OR "miRNA, Primary"[Title/Abstract] OR "pri-miRNA"[Title/Abstract] OR "pri miRNA"[Title/Abstract] OR "RNA, Small Temporal"[Title/Abstract] OR "Temporal RNA, Small"[Title/Abstract] OR stRNA[Title/Abstract] OR "Small Temporal RNA"[Title/Abstract] OR "pre-miRNA"[Title/Abstract] OR "pre miRNA"[Title/Abstract])) OR ((Epigenomics[MeSH Terms]) OR (Epigenomics[Title/Abstract] OR Epigenomic[Title/Abstract] OR Epigenetics[Title/Abstract] OR Epigenetic[Title/Abstract]))) AND (((Cisplatin[MeSH Terms]) OR (Cisplatin[Title/Abstract] OR "cis-Diamminedichloroplatinum(II)"[Title/Abstract] OR "Platinum Diamminodichloride"[Title/Abstract] OR "Diamminodichloride, Platinum"[Title/Abstract] OR "cis-Platinum"[Title/Abstract] OR "cis Platinum"[Title/Abstract] OR "Dichlorodiammineplatinum"[Title/Abstract] OR "cis-Diamminedichloroplatinum"[Title/Abstract] OR "cis Diamminedichloroplatinum"[Title/Abstract] OR "cis-Dichlorodiammineplatinum(II) "[Title/Abstract] OR NSC-119875[Title/Abstract] OR Platino[Title/Abstract] OR Platinol[Title/Abstract] OR Biocisplatinum[Title/Abstract] OR Platidiam[Title/Abstract])) OR ((Platinum Compounds[MeSH Terms]) OR ("Platinum Compounds"[Title/Abstract] OR "Compounds, Platinum"[Title/Abstract] OR "Plantinum-Containing Compounds"[Title/Abstract] OR "Compounds, Plantinum-Containing"[Title/Abstract] OR "Plantinum Containing Compounds"[Title/Abstract] OR "Platinum Compounds, Inorganic"[Title/Abstract] OR "Compounds, Inorganic Platinum"[Title/Abstract] OR "Inorganic Platinum Compounds"[Title/Abstract])))) AND ((((((Kidney[MeSH Terms]) OR (Kidney[Title/Abstract] OR Kidneys[Title/Abstract])) OR ((Acute Kidney Injury[MeSH Terms]) OR ("Acute Kidney Injury"[Title/Abstract] OR "Acute Kidney Injuries"[Title/Abstract] OR "Kidney Injuries, Acute"[Title/Abstract] OR "Kidney Injury, Acute"[Title/Abstract] OR "Acute Renal Injury"[Title/Abstract] OR "Acute Renal Injuries"[Title/Abstract] OR "Renal Injuries, Acute"[Title/Abstract] OR "Renal Injury, Acute"[Title/Abstract] OR "Renal Insufficiency, Acute"[Title/Abstract] OR "Acute Renal Insufficiencies"[Title/Abstract] OR "Renal Insufficiencies, Acute"[Title/Abstract] OR "Acute Renal Insufficiency"[Title/Abstract] OR "Kidney Insufficiency, Acute"[Title/Abstract] OR "Acute Kidney Insufficiencies"[Title/Abstract] OR "Kidney Insufficiencies, Acute"[Title/Abstract] OR "Acute Kidney Insufficiency"[Title/Abstract] OR "Kidney Failure, Acute"[Title/Abstract] OR "Acute Kidney Failures"[Title/Abstract] OR "Kidney Failures, Acute"[Title/Abstract] OR "Acute Renal Failure"[Title/Abstract] OR "Acute Renal Failures"[Title/Abstract] OR "Renal Failures, Acute"[Title/Abstract] OR "Renal Failure, Acute"[Title/Abstract] OR "Acute Kidney Failure"[Title/Abstract]))) OR ((Renal Insufficiency, Chronic[MeSH Terms]) OR ("Renal Insufficiency, Chronic"[Title/Abstract] OR "Chronic Renal Insufficiencies"[Title/Abstract] OR "Renal Insufficiencies, Chronic"[Title/Abstract] OR "Chronic Renal Insufficiency"[Title/Abstract] OR "Kidney Insufficiency, Chronic"[Title/Abstract] OR "Chronic Kidney Insufficiency"[Title/Abstract] OR "Chronic Kidney Insufficiencies"[Title/Abstract] OR "Kidney Insufficiencies, Chronic"[Title/Abstract] OR "Chronic Kidney Diseases"[Title/Abstract] OR "Chronic Kidney Disease"[Title/Abstract] OR "Disease,

Chronic Kidney"[Title/Abstract] OR "Diseases, Chronic Kidney"[Title/Abstract] OR "Kidney Disease, Chronic"[Title/Abstract] OR "Kidney Diseases, Chronic"[Title/Abstract] OR "Chronic Renal Diseases"[Title/Abstract] OR "Chronic Renal Disease"[Title/Abstract] OR "Disease, Chronic Renal"[Title/Abstract] OR "Diseases, Chronic Renal"[Title/Abstract] OR "Renal Disease, Chronic"[Title/Abstract] OR "Renal Diseases, Chronic"[Title/Abstract]))) OR (Nephrotoxicity)) OR ("kidney toxicity" OR "nephro toxicity" OR "renal toxicity"))

Total of articles recovered: 84

B. PUBMED PMC

((((MicroRNAs[MeSH Terms]) OR (MicroRNAs[Title/Abstract] OR "MicroRNA"[Title/Abstract] OR miRNAs[Title/Abstract] OR "Micro RNA"[Title/Abstract] OR "RNA, Micro"[Title/Abstract] OR miRNA[Title/Abstract] OR "Primary MicroRNA"[Title/Abstract] OR "MicroRNA, Primary"[Title/Abstract] OR "Primary miRNA"[Title/Abstract] OR "miRNA, Primary"[Title/Abstract] OR "pri-miRNA"[Title/Abstract] OR "pri miRNA"[Title/Abstract] OR "RNA, Small Temporal"[Title/Abstract] OR "Temporal RNA, Small"[Title/Abstract] OR stRNA[Title/Abstract] OR "Small Temporal RNA"[Title/Abstract] OR "pre-miRNA"[Title/Abstract] OR "pre miRNA"[Title/Abstract])) OR ((Epigenomics[MeSH Terms]) OR (Epigenomics[Title/Abstract] OR Epigenomic[Title/Abstract] OR Epigenetics[Title/Abstract] OR Epigenetic[Title/Abstract]))) AND (((Cisplatin[MeSH Terms]) OR (Cisplatin[Title/Abstract] OR "cis-Diamminedichloroplatinum(II)"[Title/Abstract] OR "Platinum Diamminodichloride"[Title/Abstract] OR "Diamminodichloride, Platinum"[Title/Abstract] OR "cis-Platinum"[Title/Abstract] OR "cis Platinum"[Title/Abstract] OR "Dichlorodiammineplatinum"[Title/Abstract] OR "cis-Diamminedichloroplatinum"[Title/Abstract] OR "cis Diamminedichloroplatinum"[Title/Abstract] OR "cis-Dichlorodiammineplatinum(II) "[Title/Abstract] OR NSC-119875[Title/Abstract] OR Platino[Title/Abstract] OR Platinol[Title/Abstract] OR Biocisplatinum[Title/Abstract] OR Platidiam[Title/Abstract])) OR ((Platinum Compounds[MeSH Terms]) OR ("Platinum Compounds"[Title/Abstract] OR "Compounds, Platinum"[Title/Abstract] OR "Plantinum-Containing Compounds"[Title/Abstract] OR "Compounds, Plantinum-Containing"[Title/Abstract] OR "Plantinum Containing Compounds"[Title/Abstract] OR "Platinum Compounds, Inorganic"[Title/Abstract] OR "Compounds, Inorganic Platinum"[Title/Abstract] OR "Inorganic Platinum Compounds"[Title/Abstract])))) AND ((((((Kidney[MeSH Terms]) OR (Kidney[Title/Abstract] OR Kidneys[Title/Abstract])) OR ((Acute Kidney Injury[MeSH Terms]) OR ("Acute Kidney Injury"[Title/Abstract] OR "Acute Kidney Injuries"[Title/Abstract] OR "Kidney Injuries, Acute"[Title/Abstract] OR "Kidney Injury, Acute"[Title/Abstract] OR "Acute Renal Injury"[Title/Abstract] OR "Acute Renal Injuries"[Title/Abstract] OR "Renal Injuries, Acute"[Title/Abstract] OR "Renal Injury, Acute"[Title/Abstract] OR "Renal Insufficiency, Acute"[Title/Abstract] OR "Acute Renal Insufficiencies"[Title/Abstract] OR "Renal Insufficiencies, Acute"[Title/Abstract] OR "Acute Renal Insufficiency"[Title/Abstract] OR "Kidney Insufficiency, Acute"[Title/Abstract] OR "Acute Kidney Insufficiencies"[Title/Abstract] OR "Kidney Insufficiencies, Acute"[Title/Abstract] OR "Acute Kidney Insufficiency"[Title/Abstract] OR "Kidney Failure, Acute"[Title/Abstract] OR "Acute Kidney Failures"[Title/Abstract] OR "Kidney Failures, Acute"[Title/Abstract] OR "Acute Renal Failure"[Title/Abstract] OR "Acute Renal Failures"[Title/Abstract] OR "Renal Failures, Acute"[Title/Abstract] OR "Renal Failure,

Acute"[Title/Abstract] OR "Acute Kidney Failure"[Title/Abstract]))) OR ((Renal Insufficiency, Chronic[MeSH Terms]) OR ("Renal Insufficiency, Chronic"[Title/Abstract] OR "Chronic Renal Insufficiencies"[Title/Abstract] OR "Renal Insufficiencies, Chronic"[Title/Abstract] OR "Chronic Renal Insufficiency"[Title/Abstract] OR "Kidney Insufficiency, Chronic"[Title/Abstract] OR "Chronic Kidney Insufficiency"[Title/Abstract] OR "Chronic Kidney Insufficiencies"[Title/Abstract] OR "Kidney Insufficiencies, Chronic"[Title/Abstract] OR "Chronic Kidney Diseases"[Title/Abstract] OR "Chronic Kidney Disease"[Title/Abstract] OR "Disease, Chronic Kidney"[Title/Abstract] OR "Diseases, Chronic Kidney"[Title/Abstract] OR "Kidney Disease, Chronic"[Title/Abstract] OR "Kidney Diseases, Chronic"[Title/Abstract] OR "Chronic Renal Diseases"[Title/Abstract] OR "Chronic Renal Disease"[Title/Abstract] OR "Disease, Chronic Renal"[Title/Abstract] OR "Diseases, Chronic Renal"[Title/Abstract] OR "Renal Disease, Chronic"[Title/Abstract] OR "Renal Diseases, Chronic"[Title/Abstract]))) OR (Nephrotoxicity)) OR ("kidney toxicity" OR "nephro toxicity" OR "renal toxicity"))

Total of articles recovered: 39

C. BIREME

micrornas OR micrornas OR microarns OR epigenômica OR epigenomics OR epigenómica) AND (cisplatino OR cisplatin OR cisplatino OR "Compostos de Platina" OR "Platinum Compounds" OR "Compuestos de Platino") AND (rim OR kidney OR riñón OR "Lesão Renal Aguda" OR "Acute Kidney Injury" OR "Lesión Renal Aguda" OR "Insuficiência Renal Crônica" OR "Renal Insufficiency, Chronic" OR "Insuficiencia Renal Crónica" OR nephrotoxicity OR "kidney toxicity" OR "nephro toxicity" OR "renal toxicity")

Total of articles recovered: 107

D. WEB OF SCIENCE

TOPIC: (MicroRNAs OR "MicroRNA" OR miRNAs OR "Micro RNA" OR "RNA, Micro" OR miRNA OR "Primary MicroRNA" OR "MicroRNA, Primary" OR "Primary miRNA" OR "miRNA, Primary" OR "pri-miRNA" OR "pri miRNA" OR "RNA, Small Temporal" OR "Temporal RNA, Small" OR stRNA OR "Small Temporal RNA" OR "pre-miRNA" OR "pre miRNA") OR TOPIC: (Epigenomics OR Epigenomic OR Epigenetics OR Epigenetic) Indexes=SCI-EXPANDED, SSCI, A&HCI, CPCI-S, CPCI-SSH, ESCI Timespan=All years AND TOPIC: (Cisplatin OR "cis-iamminedichloroplatinum(II) " OR "Platinum Diamminodichloride" OR "Diamminodichloride, Platinum" OR "cis-Platinum" OR "cis Platinum" OR "Dichlorodiammineplatinum" OR "cis-Diamminedichloroplatinum" OR "cis Diamminedichloroplatinum" OR "cis-Dichlorodiammineplatinum(II) " OR NSC-119875 OR Platino OR Platinol OR Biocisplatinum OR Platidiam) OR TOPIC: ("Platinum Compounds" OR "Compounds, Platinum" OR "Plantinum-Containing Compounds" OR "Compounds, Plantinum-Containing" OR "Plantinum Containing Compounds" OR "Platinum Compounds, Inorganic" OR "Compounds, Inorganic Platinum" OR "Inorganic Platinum Compounds") Indexes=SCI-EXPANDED, SSCI, A&HCI, CPCI-S, CPCI-SSH, SCI Timespan=All years AND TOPIC: (Kidney OR Kidneys) OR TOPIC: ("Acute Kidney Injury" OR "Acute Kidney Injuries" OR "Kidney Injuries, Acute" OR "Kidney Injury, Acute" OR "Acute Renal Injury" OR "Acute Renal Injuries" OR "Renal Injuries, Acute" OR "Renal Injury, Acute" OR "Renal Insufficiency, Acute" OR "Acute Renal Insufficiencies" OR "Renal Insufficiencies, Acute"

OR "Acute Renal Insufficiency" OR "Kidney Insufficiency, Acute" OR "Acute Kidney Insufficiencies" OR "Kidney Insufficiencies, Acute" OR "Acute Kidney Insufficiency" OR "Kidney Failure, Acute" OR "Acute Kidney Failures" OR "Kidney Failures, Acute" OR "Acute Renal Failure" OR "Acute Renal Failures" OR "Renal Failures, Acute" OR "Renal Failure, Acute" OR "Acute Kidney Failure") OR TOPIC: ("Renal Insufficiency, Chronic" OR "Chronic Renal Insufficiencies" OR "Renal Insufficiencies, Chronic" OR "Chronic Renal Insufficiency" OR "Kidney Insufficiency, Chronic" OR "Chronic Kidney Insufficiency" OR "Chronic Kidney Insufficiencies" OR "Kidney Insufficiencies, Chronic" OR "Chronic Kidney Diseases" OR "Chronic Kidney Disease" OR "Disease, Chronic Kidney" OR "Diseases, Chronic Kidney" OR "Kidney Disease, Chronic" OR "Kidney Diseases, Chronic" OR "Chronic Renal Diseases" OR "Chronic Renal Disease" OR "Disease, Chronic Renal" OR "Diseases, Chronic Renal" OR "Renal Disease, Chronic" OR "Renal Diseases, Chronic") OR All Fields: (Nephrotoxicity OR "kidney toxicity" OR "nephro toxicity" OR "renal toxicity") Indexes=SCI-EXPANDED, SSCI, A&HCI, CPCI-S, CPCI-SSH, ESCI Timespan=All years

Total of articles recovered: 108

E. SCOPUS

(TITLE-ABS-KEY (micrornas OR "MicroRNA" OR mirnas OR "Micro RNA" OR "RNA, Micro" OR mirna OR "Primary MicroRNA" OR "MicroRNA, Primary" OR "Primary miRNA" OR "miRNA, Primary" OR "pri-miRNA" OR "pri miRNA" OR "RNA, Small Temporal" OR "Temporal RNA, Small" OR strna OR "Small Temporal RNA" OR "pre-miRNA" OR "pre miRNA") OR TITLE-ABS-KEY (epigenomics OR epigenomic OR epigenetics OR epigenetic)) AND (TITLE-ABS-KEY (cisplatin OR "cis-Diamminedichloroplatinum(II)" OR "Platinum Diamminodichloride" OR "Diamminodichloride, Platinum" OR "cis-Platinum" OR "cis Platinum" OR "Dichlorodiammineplatinum" OR "cis-Diamminedichloroplatinum" OR "cis Diamminedichloroplatinum" OR "cis-Dichlorodiammineplatinum(II) " OR nsc-119875 OR platino OR platinol OR biocisplatinum OR platidiam) OR TITLE-ABS-KEY ("Platinum Compounds" OR "Compounds, Platinum" OR "Plantinum-Containing Compounds" OR "Compounds, Plantinum-Containing" OR "Plantinum Containing Compounds" OR "Platinum Compounds, Inorganic" OR "Compounds, Inorganic Platinum" OR "Inorganic Platinum Compounds")) AND (TITLE-ABS-KEY (kidney OR kidneys) OR TITLE-ABS-KEY ("Acute Kidney Injury" OR "Acute Kidney Injuries" OR "Kidney Injuries, Acute" OR "Kidney Injury, Acute" OR "Acute Renal Injury" OR "Acute Renal Injuries" OR "Renal Injuries, Acute" OR "Renal Injury, Acute" OR "Renal Insufficiency, Acute" OR "Acute Renal Insufficiencies" OR "Renal Insufficiencies, Acute" OR "Acute Renal Insufficiency" OR "Kidney Insufficiency, Acute" OR "Acute Kidney Insufficiencies" OR "Kidney Insufficiencies, Acute" OR "Acute Kidney Insufficiency" OR "Kidney Failure, Acute" OR "Acute Kidney Failures" OR "Kidney Failures, Acute" OR "Acute Renal Failure" OR "Acute Renal Failures" OR "Renal Failures, Acute" OR "Renal Failure, Acute" OR "Acute Kidney Failure") OR TITLE-ABS-KEY ("Renal Insufficiency, Chronic" OR "Chronic Renal Insufficiencies" OR "Renal Insufficiencies, Chronic" OR "Chronic Renal Insufficiency" OR "Kidney Insufficiency, Chronic" OR "Chronic Kidney Insufficiency" OR "Chronic Kidney Insufficiencies" OR "Kidney Insufficiencies, Chronic" OR "Chronic Kidney Diseases" OR "Chronic Kidney Disease" OR "Disease, Chronic Kidney" OR "Diseases, Chronic Kidney" OR "Kidney Disease, Chronic" OR "Kidney Diseases, Chronic" OR "Chronic Renal Diseases" OR "Chronic Renal Disease" OR "Disease, Chronic Renal" OR "Diseases, Chronic

Renal" OR "Renal Disease, Chronic" OR "Renal Diseases, Chronic") OR ALL (nephrotoxicity OR "kidney toxicity" OR "nephro toxicity" OR "renal toxicity"))

Total of articles recovered: 303

F. EMBASE

('microrna'/exp OR 'microrna'/syn OR 'epigenetics'/exp OR 'epigenetics'/syn) AND ('cisplatin'/exp OR 'cisplatin'/syn OR 'platinum derivative'/exp OR 'platinum derivative'/syn) AND ('kidney'/exp OR 'kidney'/syn OR 'acute kidney failure'/exp OR 'acute kidney failure'/syn OR 'chronic kidney failure'/exp OR 'chronic kidney failure'/syn OR 'nephrotoxicity'/exp OR 'nephrotoxicity'/syn)

Total of articles recovered: 250

G. COCHRANE

MeSH descriptor: [MicroRNAs] explode all trees OR (MicroRNAs OR "MicroRNA" OR miRNAs OR "Micro RNA" OR "RNA, Micro" OR miRNA OR "Primary MicroRNA" OR "MicroRNA, Primary" OR "Primary miRNA" OR "miRNA, Primary" OR "pri-miRNA" OR "pri miRNA" OR "RNA, Small Temporal" OR "Temporal RNA, Small" OR stRNA OR "Small Temporal RNA" OR "pre-miRNA" OR "pre miRNA"):ti,ab,kw OR MeSH descriptor: [Epigenomics] in all MeSH products OR (Epigenomics OR Epigenomic OR Epigenetics OR Epigenetic):ti,ab,kw AND MeSH descriptor: [Cisplatin] explode all trees OR (Cisplatin OR "cis-Diamminedichloroplatinum(II)" OR "Platinum Diamminodichloride" OR "Diamminodichloride, Platinum" OR "cis-Platinum" OR "cis Platinum" OR "Dichlorodiammineplatinum" OR "cis-Diamminedichloroplatinum" OR "cis Diamminedichloroplatinum" OR "cis-Dichlorodiammineplatinum(II) " OR NSC-119875 OR Platino OR Platinol OR Biocisplatinum OR Platidiam):ti,ab,kw OR MeSH descriptor: [Platinum Compounds] explode all trees OR ("Platinum Compounds" OR "Compounds, Platinum" OR "Plantinum-Containing Compounds" OR "Compounds, Plantinum-Containing" OR "Plantinum Containing Compounds" OR "Platinum Compounds, Inorganic" OR "Compounds, Inorganic Platinum" OR "Inorganic Platinum Compounds"):ti,ab,kw AND MeSH descriptor: [Kidney] explode all trees OR MeSH descriptor: [Kidney] explode all trees OR MeSH descriptor: [Acute Kidney Injury] explode all trees OR ("Acute Kidney Injury" OR "Acute Kidney Injuries" OR "Kidney Injuries, Acute" OR "Kidney Injury, Acute" OR "Acute Renal Injury" OR "Acute Renal Injuries" OR "Renal Injuries, Acute" OR "Renal Injury, Acute" OR "Renal Insufficiency, Acute" OR "Acute Renal Insufficiencies" OR "Renal Insufficiencies, Acute" OR "Acute Renal Insufficiency" OR "Kidney Insufficiency, Acute" OR "Acute Kidney Insufficiencies" OR "Kidney Insufficiencies, Acute" OR "Acute Kidney Insufficiency" OR "Kidney Failure, Acute" OR "Acute Kidney Failures" OR "Kidney Failures, Acute" OR "Acute Renal Failure" OR "Acute Renal Failures" OR "Renal Failures, Acute" OR "Renal Failure, Acute" OR "Acute Kidney Failure"):ti,ab,kw OR MeSH descriptor: [Renal Insufficiency, Chronic] explode all trees OR ("Renal Insufficiency, Chronic" OR "Chronic Renal Insufficiencies" OR "Renal Insufficiencies, Chronic" OR "Chronic Renal Insufficiency" OR "Kidney Insufficiency, Chronic" OR "Chronic Kidney Insufficiency" OR "Chronic Kidney Insufficiencies" OR "Kidney Insufficiencies, Chronic" OR "Chronic Kidney Diseases" OR "Chronic Kidney Disease" OR "Disease, Chronic Kidney" OR "Diseases, Chronic Kidney" OR "Kidney Disease, Chronic" OR "Kidney Diseases, Chronic" OR "Chronic Renal

Diseases" OR "Chronic Renal Disease" OR "Disease, Chronic Renal" OR "Diseases, Chronic Renal" OR "Renal Disease, Chronic" OR "Renal Diseases, Chronic"):ti,ab,kw OR (Nephrotoxicity OR "kidney toxicity" OR "nephro toxicity" OR "renal toxicity")

Total of articles recovered: 3

H. EBSCOHOST

((MicroRNAs OR "MicroRNA" OR miRNAs OR "Micro RNA" OR "RNA, Micro" OR miRNA OR "Primary MicroRNA" OR "MicroRNA, Primary" OR "Primary miRNA" OR "miRNA, Primary" OR "pri-miRNA" OR "pri miRNA" OR "RNA, Small Temporal" OR "Temporal RNA, Small" OR stRNA OR "Small Temporal RNA" OR "pre-miRNA" OR "pre miRNA") OR (Epigenomics OR Epigenomic OR Epigenetics OR Epigenetic)) AND ((Cisplatin OR "cis-Diamminedichloroplatinum(II)" OR "Platinum Diamminodichloride" OR "Diamminodichloride, Platinum" OR "cis-Platinum" OR "cis Platinum" OR "Dichlorodiammineplatinum" OR "cis-Diamminedichloroplatinum" OR "cis Diamminedichloroplatinum" OR "cis-Dichlorodiammineplatinum(II) " OR NSC-119875 OR Platino OR Platinol OR Biocisplatinum OR Platidiam) OR ("Platinum Compounds" OR "Compounds, Platinum" OR "Plantinum-Containing Compounds" OR "Compounds, Plantinum-Containing" OR "Plantinum Containing Compounds" OR "Platinum Compounds, Inorganic" OR "Compounds, Inorganic Platinum" OR "Inorganic Platinum Compounds")) AND ((Kidney OR Kidneys) OR ("Acute Kidney Injury" OR "Acute Kidney Injuries" OR "Kidney Injuries, Acute" OR "Kidney Injury, Acute" OR "Acute Renal Injury" OR "Acute Renal Injuries" OR "Renal Injuries, Acute" OR "Renal Injury, Acute" OR "Renal Insufficiency, Acute" OR "Acute Renal Insufficiencies" OR "Renal Insufficiencies, Acute" OR "Acute Renal Insufficiency" OR "Kidney Insufficiency, Acute" OR "Acute Kidney Insufficiencies" OR "Kidney Insufficiencies, Acute" OR "Acute Kidney Insufficiency" OR "Kidney Failure, Acute" OR "Acute Kidney Failures" OR "Kidney Failures, Acute" OR "Acute Renal Failure" OR "Acute Renal Failures" OR "Renal Failures, Acute" OR "Renal Failure, Acute" OR "Acute Kidney Failure") OR ("Renal Insufficiency, Chronic" OR "Chronic Renal Insufficiencies" OR "Renal Insufficiencies, Chronic" OR "Chronic Renal Insufficiency" OR "Kidney Insufficiency, Chronic" OR "Chronic Kidney Insufficiency" OR "Chronic Kidney Insufficiencies" OR "Kidney Insufficiencies, Chronic" OR "Chronic Kidney Diseases" OR "Chronic Kidney Disease" OR "Disease, Chronic Kidney" OR "Diseases, Chronic Kidney" OR "Kidney Disease, Chronic" OR "Kidney Diseases, Chronic" OR "Chronic Renal Diseases" OR "Chronic Renal Disease" OR "Disease, Chronic Renal" OR "Diseases, Chronic Renal" OR "Renal Disease, Chronic" OR "Renal Diseases, Chronic") OR (Nephrotoxicity))

Total of articles recovered: 108
