# Supplementary material for: Dysregulated MicroRNAs as Biomarkers or Therapeutic Targets in Cisplatin-Induced Nephrotoxicity: A Systematic Review
Source: Int J Mol Sci. 2021 Nov 25;22(23):12765. doi: 10.3390/ijms222312765 (PMC8657822; doi:10.3390/ijms222312765)
Supplement: Supplementary file 1 [file ijms-22-12765-s001.zip › 4 - Supplementary Figures S1 and S2.pdf]

Supplementary Figures S1 and S2

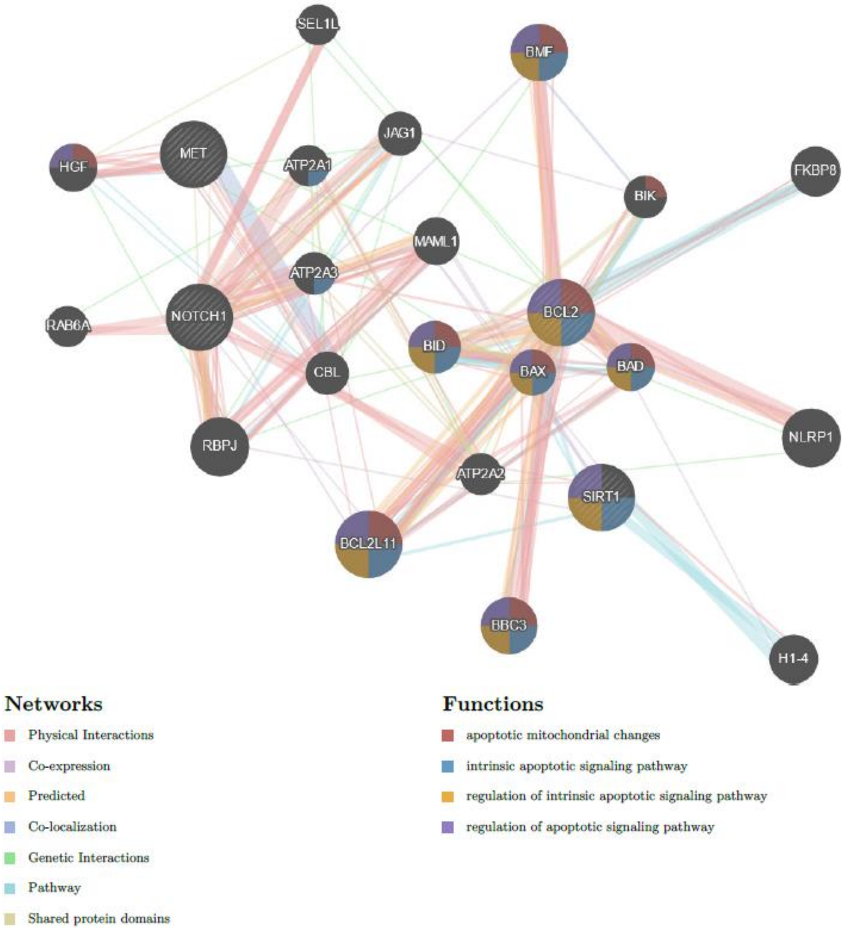

Figure S1. Pathways related to the main miR-34a targets (*BCL2*, *MET*, *NOTCH1*, and *SIRT1*), selected from *in silico* analysis (the figure was generated using the GeneMANIA website tool, https://genemania.org/).

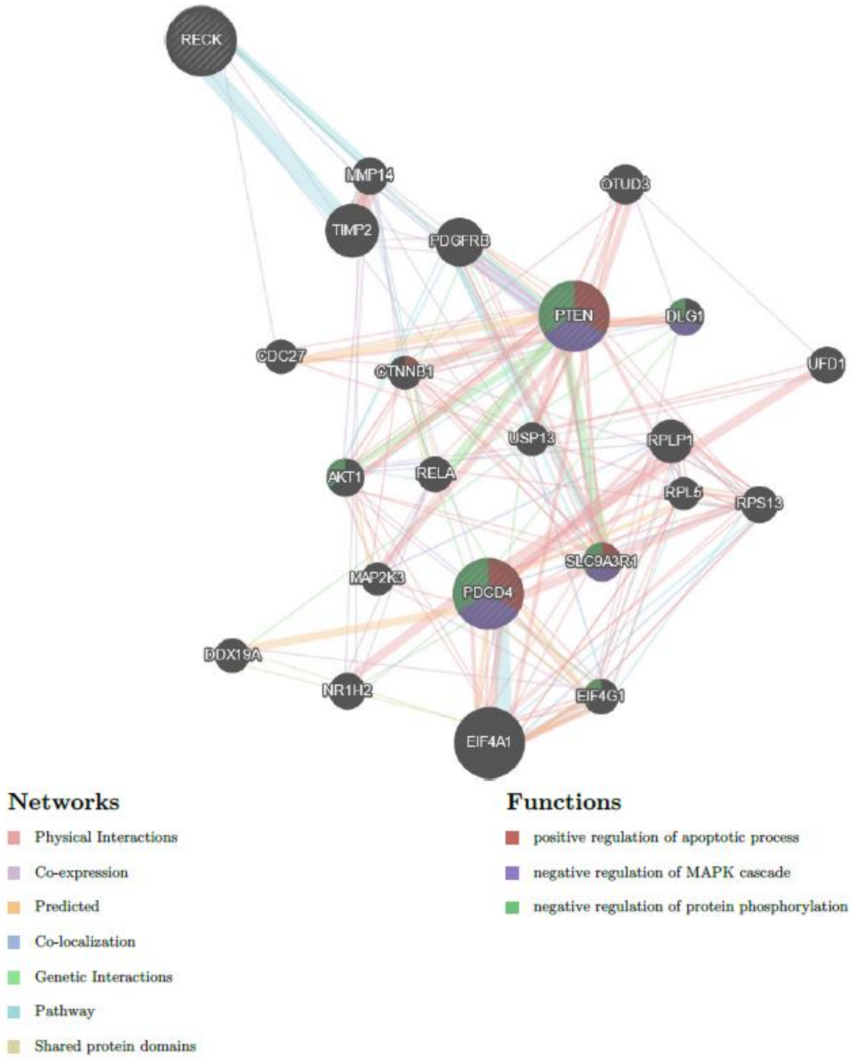

Figure S2. Pathways related to the main miR-21 targets (*PDCD4*, *PTEN*, and *RECK*), selected from *in silico* analysis (the figure was generated using the GeneMANIA website tool, https://genemania.org/).
